# Characterizing Molecular Mechanisms of Imidacloprid Resistance in Select Populations of *Leptinotarsa decemlineata* in the Central Sands Region of Wisconsin

**DOI:** 10.1371/journal.pone.0147844

**Published:** 2016-01-28

**Authors:** Justin Clements, Sean Schoville, Nathan Peterson, Que Lan, Russell L. Groves

**Affiliations:** Department of Entomology, University of Wisconsin-Madison, Madison, Wisconsin, United States of America; Federal University of Viçosa, BRAZIL

## Abstract

The Colorado potato beetle, *Leptinotarsa decemlineata* (Say), is a major agricultural pest in the Central Sands region of Wisconsin. Imidacloprid, a neonicotinoid insecticide, has commonly been used for control of *L*. *decemlineata* since its registration in 1995. In the last 10 years, many field populations of *L*. *decemlineata* have begun to show increasing imidacloprid resistance. We studied resistance phenotype as a phenomenon that reduces neonicotinoid efficacy and has practical consequences for potato pest management. Although we have not observed complete field failure following the use of these products, multiple studies have demonstrated that the lethal concentration to kill 50% of the test organisms (LC_50_) in different field populations of *L*. *decemlineata* varies greatly which may suggest that resistance of *L*. *decemlineata* is heritable and involves genetic changes. An important challenge in understanding resistance is assessing the genetic mechanisms associated with resistance and classifying up-regulated genes that may be involved in combating an insecticide insult. In this study we uncovered trends in imidacloprid phenotypic response that have developed in the region by estimating the LC_50_ values among different field populations against a range of imidacloprid doses. The LC_50_ values collected in 2008–2011, and more recently in 2013 and 2014, show that some field locations remain susceptible to imidacloprid, while nearby fields (<100km) have developed high levels of resistance. We also sought to uncover potential mechanisms of resistance at each field location. We compiled a transcriptome for populations, characterized as phenotypically ‘susceptible’ and ‘resistant’, by isolating mRNA from adult beetles and analyzing gene expression level differences. Strong differences were observed in constituently up and down-regulated genes among different field populations. Most significantly, the up-regulation of 3 cytochrome p450s and a glutathione synthetase related protein in multiple resistant populations provide a mechanistic explanation of resistance evolution in *L*. *decemlineata*.

## Introduction

The Colorado potato beetle (CPB), *Leptinotarsa decemlineata* (Say), is a key agricultural pest infesting commercial potato (*Solanum tuberosum*), as well as tomatoes (*S*. *lypcopersicum*), eggplants (*S*. *melongena*) and peppers (*S*. *annuum)* [1]. *L*. *decemlineata* is thought to have expanded from the southwestern United States and Mexico to a global range encompassing the United States, Mexico, Europe, and Asia, inclusive of over 16 million km^2^, and switching from its native host plant buffalobur (*Solanum rostratum)* to agricultural crops [1,2]. With very few natural enemies, the beetles can be devastating on crops if an insecticide treatment is not used. Adult beetles consume as much as 10 cm^2^ of foliage daily [2], leading to 50–100% crop loss in unmanaged situations. Potato fields throughout the United States are regularly treated with a combination of at-plant, systemic and foliar applications of neonicotinoid insecticides throughout the growing season to control *L*. *decemlineata*. Since its initial registration in 1995, Imidacloprid has been the most widely used neonicotinoid insecticide for crop protection. Imidacloprid affects the nicotinic acetylcholine receptor (nAChR) in insects and causes the accumulation of acetylcholine at nerve synapses, which ultimately leads to paralysis and death of the target organism [3]. Due to its unique structure, imidacloprid has high specificity to insect nAChR and low specificity to vertebrate nAChR, leading to low mammalian toxicity [3] and permitting a variety of uses on many crop and non-crop plant species.

A number of studies have shown that select populations of *L*. *decemlineata* are developing resistance to neonicotinoid insecticides, including field populations in Wisconsin, Maine, Michigan and New York [2,4–7]. Resistant populations of beetles are typically classified using LC_50_ assays, where the estimated ratio (RR) is calculated as the ratio of the estimated LC_50_ of the test population over the LC_50_ of a reference susceptible population [8]. If the RR value is greater than 10, a population is considered to have (or to be trending towards) a resistant phenotype. Measured levels of imidacloprid resistance have increased in select populations of *L*. *decemlineata* from the Central Sands region of Wisconsin since 2008 [4]. This is similar to observations of imidacloprid resistance found in field populations of *L*. *decemlineata* from other states [9]. While RR values are only a snapshot of a population’s resistance phenotype, a similar study from 2011 revealed large increases in resistance in field populations of *L*. *decemlineata* in Michigan over consecutive years [9]. Increasing levels of observed resistance have also coincided with an increasing frequency of neonicotinoid applications to obtain effective control of *L*. *decemlineata* populations, evidenced by higher LC_50_ values in commercial potato fields using imidacloprid [4], which may further exacerbate the potential for increased resistance. Understanding the population dynamics and underlining genetic resistance mechanisms that give rise to a resistant phenotype in *L*. *decemlineata* is of critical importance in managing beetle populations. With an improved understanding of the genetic mechanisms that play a role in *L*. *decemlineata* resistance development, we will be able to better monitor field populations for resistance and may also have the capacity to predict the spread and prevalence of resistance in the future.

Previous studies have suggested that *L*. *decemlineata*, as well as other insects, possess four candidate mechanisms of resistance to toxic chemicals [2,10]. The first is described as target site insensitivity. In the case of neonicotinoid resistance, this would involve a mutation in the nAChR, similar to that which has been found in the brown planthopper [11]. A second mechanism is enhanced metabolic breakdown, whereby increased metabolism by enzymes purge cells of toxic compounds. Proteins such as monooxygenases, which include the cytochrome p450s, are primary agents (Phase I metabolism) enhancing metabolic breakdown. cytochrome p450s have been shown to play a vital role in insect metabolism of imidacloprid [12,13] as well as other neonicotinoid insecticides. Other secondary agents (Phase 2 metabolism) include enzymes such as glutathione S-transferases [12] which conjugate neonicotinoid molecules to increase water solubility and facilitate bodily excretion. The third potential mechanism is increased excretion, which can also be performed by some metabolic mechanisms or reduced penetration which would inhibit the pesticide from reaching its target site. This can be accomplished by a suite of cellular mechanisms. The fourth mechanism is simply classified as behavioral resistance, whereby beetles avoid exposure to pesticide by modifying their phenology, foraging behavior, or habitat choices, avoiding leaves with higher concentrations of pesticide and even avoidance of areas that have been treated [2]. These four mechanisms can be classified into two categories of protection: toxicokinetic and toxicodynamic mechanisms [14]. Toxicokinetic mechanisms play a role in absorption of the insecticide into the organism and to the fate of the insecticide after it enters the organism (biotransformation and excretion), while toxicodynamic mechanisms involve the interactions of the pesticides with their target sites [14]. These interactions can be complex in nature and multiple interactions can take place simultaneously.

In the current study, our primary aim was to uncover any genes that were involved in enhanced molecular breakdown or reduced penetration of imidacloprid. Specifically, we attempted to determine if genes in resistant populations were constitutively active or were differently expressed compared to a susceptible reference population. We composed a transcriptome of all the genes expressed in both resistant and susceptible populations using RNA extracted from field populations of beetles collected in 2013. This approach allowed us to observe gene regulation across both the susceptible and resistant populations collected from a similar geographic region and similar time points during the growing season. Site selection for these experimental populations was based upon prior knowledge gained from earlier LC_50_ bioassays performed by Huseth and Groves [4] together with newly collected LC_50_ estimates. This approach provided a strong foundation to choose from among several resistant populations in the Central Sands Region of Wisconsin previously identified as resistant over successive growing seasons. Our results are principally focused on transcript expression in these *L*. *decemlineata* populations of the Central Sands Region of Wisconsin using RNA sequencing technologies. Expression studies were further validated with quantitative polymerase chain reaction (PCR). Based upon previous assessments of neonicotinoid resistance among populations in Wisconsin [4], we selected two candidate resistant populations and one nearby susceptible population to analyze in this study. This study uncovered many up-regulated genes in the two resistant populations that have the ability to combat insecticides in *L*. *decemlineata*. The two resistant populations showed some similarities in the up-regulated genes, but many of the up-regulated genes were differentially expressed between the two populations, suggesting that different populations of *L*. *decemlineata* may cope with insecticides in different manners.

## Materials and Methods

### Ethics Statement

No specific permits were required for field collection of *L*. *decemlineata* for the study described here. Access to all field sites was granted by landholders.

### *Leptinotarsa decemlineata* Collection

Two generations of *L*. *decemlineata* were collected from fields in the Central Sands region of Wisconsin in the spring and summer of 2013 and 2014. Approximately 700 overwintered adult beetles were randomly chosen from newly emerged potato plants at each of the pre-selected field locations. Adult beetles were hand-collected from plants at 4 different field locations including the Hancock Agricultural Research Station (HARS, 44.120430, -89.539149), plus three nearby agricultural fields in the Central Sands region, henceforth referred to as Systemic-1, Systemic-2, and Systemic-3. An additional set of 700 adult *L*. *decemlineata* were obtained from the Arlington Agricultural Research Station (AARS, 43.315527, -89.334545), and this population was regarded as a susceptible population. All adult beetles collected from the canopy of plants at all locations did not show any symptoms of acute poisoning at the time of collection. After collection, adult beetles were additionally maintained for 72 hours on untreated potato foliage, which was grown in an insecticide-free greenhouse on the campus of the University of Wisconsin-Madison. Following the 72 hour feeding interval, a randomly selected subset of 350–400 adults were used for LC_50_ assays, and another smaller, randomly selected subset (N = 60) was used for RNA extraction and transcriptome assembly. A second collection of approximately 200 adult *L*. *decemlineata* (termed 2^nd^ generation adults) were collected from the same field locations after they had completed their first full generation and had emerged as adults from the soil in mid-July. Here again, the adult *L*. *decemlineata* were caged for 72 hours on untreated potato foliage after collection and were later used in RNA extraction and transcriptome construction.

Overwintered adult *L*. *decemlineata* were again collected from the same populations in 2014 using similar methods and used for LC_50_ assays. Due to crop rotation in the spring of 2014, overwintered adult beetles were collected in fields that were adjacent to the previously-sampled field (less than <0.8 km apart) in 2013 where adult insects had previously been collected. Because adult *L*. *decemlineata* are not known to disperse over great distances [15] from their previous field locations, adult insects collected at these adjoining field locations were considered to be from the same field populations.

### LC_50_ Assessments within Populations

In 2013, 350 to 400 overwintered adult beetles from each population were used to determine the lethal concentration required to kill 50% of the test insects (LC_50_) using serial dilutions of technical imidacloprid (Bayer, Kansas City, Mo) in acetone. Specifically, 350 to 400 adult beetles were divided into 7 or 8 groups (n = 50) and treated with a 1μl solution of imidacloprid in acetone on the first abdominal sternite. Imidacloprid concentrations ranged between 0–2 ppm and dilutions were prepared taking into consideration the specific density of acetone. A 0 ppm acetone concentration was used as a no insecticide control. Adult beetles were placed in petri dishes with fresh foliage that was changed every day for 7 days in an incubator held at 26°C, 70% humidity, and light and dark cycle of 16 hours light and 8 hours dark. After 7 days, the number of live beetles, incapacitated beetles, and dead beetles was recorded, as measured by the pencil test [6]. Briefly, adult beetles were presented with the opportunity to climb a pencil: if they could move a full body length they were considered alive, if they appeared alive, but could not move a body length then they were considered incapacitated, and if they had no movement, even after pinching their back legs with tweezers, they were considered dead. Incapacitated and dead beetles were pooled and LC_50_ values for each population were calculated. Abbott’s correction was used to normalize the data and a log_10_ probit regression analysis (PROC PROBIT, SAS Institutes) [4,16] was used to calculate the LC_50_ for each first generation population. In 2014, a similar protocol was followed, but 450–500 beetles were used to determine the LC_50_ with 10 groups (n = 50) ranging from 0-100ppm.

### RNA Extraction and Illumina Sequencing

Total RNA was extracted for RNA sequencing from beetles collected at the AARS and the systemic-1 and systemic-3 populations in 2013. The 2 systemic populations were chosen because they were found to be the most resistant in the LC_50_ assays while the AARS population served as the susceptible comparison. At least 60 healthy, adult female beetles were randomly selected from each population and used for RNA extraction. Briefly, total RNA was extracted from each beetle with Trizol (Life Technology, Grand Island, NY) and stored at -80C for later analysis. Total RNA was pooled into three biological replicates composed of 60 individuals for each population (1 replicated representing overwintered adult beetles and 2 replicates representing 2^nd^ generation beetles) and treated with TurboDNase (Life Technology, Grand Island, NY). The quality of the RNA was examined using a 2100 Bioanalyzer (Agilent Technologies, Santa Clara, CA). RNA concentrations were measured using a Nanodrop (Thermo Fisher Scientific, Waltham, MA) and 500 ng of total RNA per replicate was submitted for RNA-sequencing (Beckman Coulter Genomics, Danvers, MA). Beckman Coulter Genomics was contracted to isolate mRNA using an Illumina automated RNA-seq platform to generate 338 million high quality reads of 2X100bp. Beckman Coulter Genomics was also contracted to perform quality checks and *de novo* assembly of RNA-seq data into a whole transcriptome encompassing all populations. Assembly was performed using Trinity Bioinformatics [17,18]. Beckman Coulter used the default settings in Trinity Bioinformatics software release 2013-11-10 with an initial input of reads from Arlington, Systemic-1, and Systemic-3 with 12 CPUs to generate the transcriptome. Raw reads were uploaded to NCBI Sequence Read Archive (SRA) SRP064192. The Transcriptome Shotgun Assembly project has been deposited at DDBJ/EMBL/GenBank under the accession GEEF00000000. The version herein described is the first version, GEEF01000000. The Transcriptome was submitted following NCBI submission criteria, and the assembled transcriptome was uploaded after removal of contamination identified by blast searched against the Univec database and contigs smaller than 200 basepairs. Gene expression was calculated in units of fragments per kilobase of exon per million fragments mapped (FPKM) using Trinity Bioinformatics for each separate biological replicate.

### Annotation of Trinity Contigs

BLAST standalone [19] was used to classify Trinity assembled contigs into possible genes. Reference protein sequences (Refseq) from *Tribolium castaneum*, *Acyrthosiphon pisum*, *Anopheles gambiae*, *Drosophila melanogaster*, and *Pediculus humanus* were downloaded from NCBI for a total of 80,498 sequences. A reference database was created with the protein sequences to blast against the transcriptome. Using Blast standalone with BLASTx, the total unique trinity contigs in the transcriptome were compared to the reference proteins (*E* value <10^−3^). Transcripts were classified based on the NCBI nomenclature returned by BLASTx. BLASTx results were uploaded into Blast2Go [20] for further data analysis. The entire transcriptome was first analyzed and the components were mapped to the corresponding GO terms, then the annotation step was run with a cutoff of E_value_ < 1E-3, annotation cut off > 45, and GO weight >5.

### Differential Gene Expression

Transcript abundance between the putative resistant and susceptible populations in 2013 was calculated using Trinity bioinformatics software (RSEM). FPKM were calculated for each set of unique contigs in each population (1 was added to each FPKM value). Gene abundance was compared between the 2^nd^ generation populations and confirmed with qPCR. A linear regression between the two biological replicates of 2^nd^ generation for each of the three different populations was conducted along with determining the FDR (false discovery rate) (P≤0.059) to eliminate outlying data in R commander [21]. The FPKM of the biological replicates in each population were averaged and the FPKM’s between the two putative resistant and the single susceptible populations were compared. Genes with a fold change greater than 2 were considered up- or down-regulated. An enrichment analysis between the annotated transcriptome and the upregulated genes found using Trinity was run using a two-tailed FDR test with 0.05 cut-off in order to determine if any group of GO terms were differentially expressed in the up-regulated components. The up-regulated GO terms were then categorized using CateGOrizer [22] with GO_slim.

### Quantitative PCR

Three biological replicates of pooled cDNA from each population from 2013 were used in cDNA synthesis for quantitative, real-time PCR (qPCR). Total RNA from each population was quantified using a Nanodrop (Thermo Fisher Scientific, Waltham, MA). DNA contamination was removed using TurboDNase (Life Technology, Grand Island, NY). DNA-free RNA was purified to remove any possibility of PCR inhibition with a MasterPure Complete DNA and RNA Purification Kit (Epicentre, Madison, WI). Following purification, RNA was suspended in 20μl of water, and the integrity was checked on a 2100 Bioanalyzer (Agilent Technologies, Santa Clara, CA). All RNA concentrations were equalized before input into the cDNA synthesis kit and the subsequent cDNA was generated with a high capacity cDNA reverse transcription kit (Applied Biosystems, Foster City, CA). The cDNA was diluted to a final concentration of 5ng/μl of RNA equivalent input for qPCR. *β*-actin was used as a reference gene. The *β*-actin primers were shortened versions previously used by Zhu [23]. The qPCR reaction was run on a CFX-96 platform (Bio-Rad Laboratories, Hercules, CA) with a master mix of Bullseye EverGreen (MIDSCI, Valley Park, MO). Genes of interest (GOI) were selected based on their relevance to this study and primers were designed to contigs found in the transcriptome. Primer and primer efficiency are found in Table 1. Primer specificity was checked against the transcriptome using BLAST. Primer efficiencies were calculated and optimized. Triplicate reactions were run at 95°C for 10min followed by 95°C for 15 s, 62°C for 60 s for 40 cycles. Data were collected for each biological replicate and relative expression of resistant strains to susceptible strains was calculated using the Pfaffl methodology [24], as seen in Eq 1. The Pfaffl methodology takes into consideration the efficiency of the primer sets and gives the ratio of the target gene to the reference population.

**Table 1:** qPCR primers and primer efficiency.

	Forward Primer (5’-3’)	Reverse Primer (5’-3’)	Primer Efficiency
B-actin (Reference)	CATCCAAGCTGTACTCTCCTTG	GGAAGAGCGTAACCTTCGTAG	1.92
Comp115309 (Cytochrome P450)	CGAGAAATGCGACCTATTCTCAG	ACACAGTCTTGGTCTTTCTTGAG	1.98
Comp105889 (Cuticular protein)	CTCCAGTGGTTCCGTTATTACAC	AGCGTAGTCGTGGAAATGTTG	1.94
Comp114026 (Glutathione synthetase)	CAGAGCAGGGTATGAACCTAATC	CCAGCCAAGTGATACTGAATCG	1.97

$$\text{ratio}=\frac{{\left(E_{\text{target}}\right)}^{\Delta {\text{CP}}_{\text{target}}\left(\text{Control}-\text{samples}\right)}}{{\left(E_{\text{reference}}\right)}^{\Delta {\text{CP}}_{\text{ref}}\left(\text{control}-\text{samples}\right)}}$$ (1)

## Results

### Topical Bioassay

Resistance (LC_50_) estimates generated in the 2013 and 2014 growing seasons closely approximate trends from previously reported data over the interval 2008–2011 in the same geographic region of the state [4]. Table 2 presents regression estimates for populations including three putative resistant populations (systemic-1, systemic-2, and systemic-3), a moderately resistant population from the HARS, and a susceptible population from the AARS over two years in the current study. The current study uses the AARS population as the reference control strain with an estimated LC_50_ value of 0.09ppm in 2013 and 4.7ppm in 2014. The field population of *L*. *decemlineata* at the AARS has never been exposed to a season-long, at-plant use of imidacloprid. As expected, the data demonstrate that the field populations at the central Wisconsin locations, which have annually received successive, at-plant neonicotinoid applications, possess higher estimated LC_50_ values, found in Table 2, and higher resistance ratios, ranging from 10.11 to 18.18 in 2013 and 1.84 to 11.16 in 2014 (a resistance ratio exceeding 10 was considered a resistant population [5] in previous investigations). The *L*. *decemlineata* populations collected from the 3 commercial potato fields in the Central Sands region had the highest overall levels of measured resistance in 2013. In 2014, only one of the field locations (systemic-3) was classified as resistant according to this designation, while the remaining two field locations had estimated resistance ratios < 10. The HARS population was classified as resistant in 2013, but also dropped below this ratio in 2014. It should be noted, however, that while resistance ratios decreased from 2013 to 2014, the LC_50_ values for all field populations substantially increased (Table 2).

**Table 2:** Regression estimates resulting from topical bioassays of adult *Leptinotarsa decemlineata* at the Arlington (AARS) and Hancock Agricultural Research Stations (HARS), plus agricultural fields classified as systemic-1, 2 and 3 in 2011, 2013 and 2014.

Population	Year^1^	N^2^	Slope(SEM)^3^	LC_50_ (PPM)	95% CI	Resistance Ratio^4^
Arlington	2011	600	3.13(0.33)	0.027	(0.028–0.34)	NA
Hancock	2011	525	1.47(0.11)	0.48	(0.4–0.6)	17.77
systemic-1	2011	425	1.63(0.14)	0.72	(0.59–0.87)	26.66
systemic-2	2011	524	1.9(0.27)	0.62	(0.41–0.94)	22.96
systemic-3	2011	500	2.03(0.73)	0.73	(0.51–1.04)	27.03
Arlington^5^	2013	400	2.23(.25)	0.09	(.08-.12)	NA
Hancock	2013	300	0.73(.23)	0.91	(.49–4.30)	10.11
systemic-1^5^	2013	350	0.69(.26)	1.83	(.21–12.36)	18.18
systemic-2	2013	350	1.49(.37)	1.10	(.58–1.61)	11.11
systemic-3^5^	2013	350	2.08(.30)	1.20	(.94–1.57)	12.12
Arlington	2014	500	0.55(.12)	4.72	(1.57–10.74)	NA
Hancock	2014	500	0.80(.09)	8.69	(5.00–15.19)	1.84
systemic-1	2014	500	1.05(1.0)	12.81	(8.73–19.96)	2.71
systemic-2	2014	500	0.71(.09)	43.67	(23.60–109.27)	9.25
systemic-3	2014	500	0.66(.09)	52.68	(25.31–154.56)	11.16

^1^ 2011 data is reported regression estimates obtained from Huseth and Groves (4).

^2^ Total number of adult beetles used in biological replicates at each location and year combination.

^3^ Mean slope estimates of the probit linearized dose response function plus standard error

^4^ Resistance ratio estimates comparing test populations to the reference control population (AARS) in each year.

^5^ Populations of *Leptinotarsa decemlineata* used in transcriptome assembly

### Transcriptome Assembly

Illumina short-read sequencing was used to sequence mRNA collected from adult female *L*. *decemlineata* and subsequently assemble a transcriptome using Trinity bioinformatics software [17,18]. A summary of data from the assembled transcriptome from all three Wisconsin *L*. *decemlineata* populations is presented in Table 3. The transcriptome was composed of RNA isolated in 2013. RNA sequencing was performed on nine samples of pooled RNA, n = 3 from each population (AARS, systemic-1 and systemic-3). The transcriptome revealed 98,002 possible unique transcripts for investigation and presumably some portion of these genes encode the aforementioned mechanisms of pesticide resistance. Subcomponents of Trinity were then grouped together and classified as individual components. From the 98,002 unique transcripts, we used 80,498 reference sequences from NCBI [25] to identify 23,860 known transcripts in our transcriptome.

**Table 3:** Summary of *Leptinotarsa decemlineata* transcriptome assembled from the 2013 beetle populations collected from the Arlington Agricultural Research Station and two field populations, termed systemic-1 and systemic-3.

Summary of *L*. *decemlineata* transcriptome	
Total assembled bases	197,128,499
Total contigs	208,754
Unique transcripts	98,002
median contig length	479 bp
Average contig	944.31 bp
NC50	1742 bp
Percent GC	36.49
Blastx hits(*e* value ≥ 0.001)	23,860

### Differential Expression Analysis

An overall goal of this study was to investigate gene expression differences between the resistant and susceptible populations. From the 23,860 transcripts identified in the analysis, we chose to focus on transcripts that encoded for genes associated with the known mechanisms of resistance. Specifically, we investigated changes in the expression levels of 8 classes of enzymes and proteins (cytochrome p450, glutathione related proteins, cuticular proteins, carboxylesterases, nicotinic acetylcholine receptors, ABC transporters, superoxide dismutase enzymes and catalase enzymes) to determine if patterns emerged in the up- or down-regulation of specific transcripts in the two resistant populations compared to the susceptible population. The transcriptome of *L*. *decemlineata* revealed 107 transcripts that encoded cytochrome p450s, 96 that encoded cuticular proteins, 21 that encoded glutathione related proteins, 16 that encoded carboxylesterases, 59 that encoded ABC transporter proteins (including multi drug resistant proteins), 7 that encoded superoxide dismutase enzymes, and 6 that encoded catalase enzymes. A portion of these may constitute possible resistance mechanisms (Table 4). The transcriptome also revealed 20 possible nAChRs transcripts, some of which are known targets of imidacloprid.

**Table 4:** Differentially expressed up- and down-regulated transcripts observed between two resistant populations compared to the Arlington Agricultural Research Station population.

	Whole Transcriptome	Systemic-1 Up-regulated	Systemic -1 Down-regulated	Systemic-3 Up-regulated	Systemic-3 Down-regulated
**Total Transcripts**	98,002	394	195	562	632
**Total Transcripts with Blastx results evaluate(0.001)**	23,860	290	78	399	405
**Cytochrome p450**	107	6	1	13	6
**Cuticular**	96	0	1	23	2
**ABC transporters**	59	4	0	7	0
**Glutathione related proteins**	21	1	0	1	0
**Nicotinic acetylcholine receptor**	20	0	0	0	0
**Catalase**	6	1	1	0	0
**Superoxide dismutase**	7	1	0	0	0
**Carboxylesterase**	16	1	0	2	0

Transcript expression was compared between the AARS susceptible population and two of the three previously described resistant populations from agricultural fields (systemic -1 and -3). For the comparison, we used two pooled samples (biological replicates) of adult *L*. *decemlineata* collected from the second generation (mid-July collections) from each field. After the transcriptome was assembled, we calculated the fragments per kilobase of exon per million fragments mapped (FPKM) of each transcript to estimate expression levels between the populations. We then identified significantly up- and down–regulated genes by adjusting levels according to a false discovery rate (FDR) of 0.059 between the populations. Gene regulation between the susceptible AARS population and the two resistant field populations (systemic-1, systemic-3) can be seen in Table 4. In the systemic-1 population there were a total of 394 transcripts, of which 290 share homology to known proteins. In the systemic-3 population, there were 562 transcripts, of which 399 share homology to known proteins. The up-regulated components of the systemic-1 and -3 populations can be seen in S1 and S2 Tables.

Using Blast2go we mapped Trinity components to Gene Ontology (GO) terms. We then classified the up- regulated genes into GO terms to examine if there was any difference in GO terms between up- regulated populations and the whole transcriptome. The analysis revealed 290 up-regulated and annotated transcripts that correlated to known genes that were classified into 349 GO terms and then grouped into 65 GO_slim classes in the systemic-1 population. The systemic-3 population had 399 up-regulated and annotated transcripts encoding for genes which were classified into 400 GO terms and were grouped in to 45 GO_slim classes. The up-regulated GO terms in both systemic-1 and -3 populations had similar GO_slim classes with 13.68% of GO terms of systemic 1 population falling into molecular functions and 16.72% of GO terms falling into biological processes. The systemic-3 population had 21.78% of GO terms falling into molecular functions and 12.34% categorized into biological processes. An enrichment analysis of the GO terms between the compiled transcriptome and the up-regulated GO terms in the systemic-1 and -3 populations showed different trends between the two resistant populations. The enrichment analysis revealed three sets of GO terms were significantly up-regulated (catalytic activity, single-organism metabolism processes, oxidoreductase activity) in the systemic-1 population, while in the systemic-3 population the enrichment analysis revealed several up-regulated GO terms including structural constituents of the cuticle, monooxygenase activity, oxidoreductase activity and oxidation-reduction processes. Significantly different GO terms (FDR > 0.05) between the up-regulated genes in the resistant populations and the entire transcriptome can be found in S3 and S4 Tables.

The up-regulated transcripts were then examined in both populations. In the up-regulated transcripts of the two resistant populations, we observed 8 shared transcripts that may play a role in insecticide resistance, including 3 cytochrome p450s, 1 carboxylesterase, 1 glutathione synthetase, and 3 proteins that are related to ABC transporters, as seen in Table 5. When we examined the fold change calculated in RSEM, we found that the 8 shared transcripts had a fold change of around two, which was the cut off for up-regulation. One of the cytochrome p450s in the systemic-3 population was up-regulated three fold. This observation could indicate that these genes are constitutively active, but not significantly over-expressed due to the fitness cost of activating multiple mechanisms of resistance simultaneously. Trends in the down-regulated transcripts were observed between the susceptible population and the two resistant populations. The two populations shared a total of 129 down-regulated transcripts. Two of these transcripts, a cytochrome p450 and a cuticular protein, encode a known mechanism of resistance.

**Table 5:** Fold changes in the up-regulated genes observed between the two resistant populations, Systemic-1 and Systemic-3.

Transcript	Systemic-1 (Fold Change)	Systemic-3 (Fold Change)	Predicted Genes from Blastx against 80,498 reference sequence^1^ in NCBI database	NCBI accession number
comp118021_c0	2.11	2.06	atp-binding cassette transporter	XP_969849
comp117821_c0	2.06	2.42	atp-binding sub-family c (cftr mrp) member 4	XP_971632
comp114343_c0	2.07	2.25	PREDICTED: similar to carboxylesterase	XP_969104
comp117371_c0	2.43	2.18	multi drug resistance 50 cg8523-pa	XP_001810982
comp114026_c0	2.08	2.66	glutathione synthetase	XP_968070
comp103658_c0	2.37	2.34	cytochrome p450 9z4	NP_001164248
comp106072_c0	2.5	2.38	cytochrome p450 9z4	NP_001164248
comp111691_c1	2.77	3.25	cytochrome p450 monooxygenase	XP_972348

^1^ Reference sequences complied from NCBI in January 2014

We then confirmed the gene regulation between the samples by using quantitative PCR (qPCR) (Table 6). From among the total up-regulated transcripts studied, we focused on transcripts that could play a role in insecticide resistance. Additionally, *β*-actin expression was used as a reference and confirmed the expression of 3 transcripts, an up-regulated cytochrome p450 in the systemic-3 population (not classified as up-regulated in systemic-1 due to an FDR value greater than 0.059), a cuticular protein that was up-regulated in the systemic-3 population, and an up-regulated glutathione synthetase protein that was also up-regulated in both resistant populations. The qPCR results in the systemic-1 and -3 populations closely resembled the fold changes calculated with FPKM.

**Table 6:** Gene expression determined by quantitative PCR.

	Arlington	Systemic-3			Systemic-1		
	Mean CT ±SD	Mean CT ±SD	FPKM Fold Change	qPCR Expression ratio	Mean CT ±SD	FPKM Fold Change	qPCR Expression ratio
ß-actin (Reference)	20.3±.30	19.39±.37	N/A	N/A	19.72±.49	N/A	N/A
comp115309 (cytochrome p450)	22.4±.38	20.04±.03	3.5	2.77	20.42±.31	2.85	2.65
comp105889 (cuticular protein)	23.9±.62	20.92±.39	6.48	3.96	22.31±1.70	1.72	1.96
comp114026 (glutathione synthetase)	27.1±1.02	24.68±.23	2.66	2.9	25.52±.39	2.08	2.04

## Discussion

The ability of *L*. *decemlineata* to become resistant to insecticides has become a major agricultural problem with wide-reaching consequences. Although previous studies have indicated that populations of *L*. *decemlineata* have developed some form of resistance to imidacloprid [2, 4–7], the molecular determinants of this resistance within or between populations of *L*. *decemlineata* remain unknown. Understanding the genetic mechanisms and whether genetic changes are shared among populations is important in predicting the evolution of resistance in other potato growing regions. Topical bioassays have been used to demonstrate resistance to neonicotinoids as early as 1997 [6]. Field populations of *L*. *decemlineata* in the Central Sands region of Wisconsin demonstrate variable levels of resistance that are maintained across years. When comparing the data between field populations collected in the same year, trends in resistance remain consistent, with treated fields having higher LC_50_ values than non-treated fields.

Although topical bioassays have considerable annual variation, with data collected in 2013 closely resembling estimates generated in a previous study [4] and data collected in 2014 resulting in much higher LC_50_ values, the trend towards increasing LC_50_ values is consistent across the field populations investigated. There is the possibility that resistance is increasing throughout Wisconsin, including at the AARS site previously considered susceptible due to its low resistance ratios and lack of pesticide exposure. However, variation in the estimated LC_50_ values is not unexpected, as subtle differences in sampling times after beetles emerged from diapause, as well as fitness costs associated with overwintering conditions, could have influenced our experimental results. Modest variations in collection times of overwintered beetles could play a large role in the fitness of the beetles and how they respond to topical bioassays. Variation between LC_50_ estimates is expected to increase as field populations of beetles become more resistant to insecticides [9]. The inter-annual variation between populations can clearly be seen as reported by Huseth and Groves [4] with some mixed populations of beetles varying in LC_50_ values as much as 20 fold between years.

A transcriptome was assembled from RNA extracted from *L*. *decemlineata* in an effort to identify the potential mechanisms of resistance. The transcriptome itself encompassed 208,754 transcripts, of which 98,002 were unique. Recently, an annotated transcriptome was published in 2014 [26] that distinguished the activity of genes in different developmental stages of *L*. *decemlineata*. This study revealed a transcriptome that contained 121,912 transcripts from both adult and larval data using 454 sequencing technology. Interestingly, they found more than twice the number of cytochrome p450 and glutathione related proteins when compared to our study (221 cytochrome p450 and 45 glutathione S-transferase proteins compared to 107 cytochrome p450 and 21 glutathione related proteins). The use of different sequencing and assembly methods, reference gene set, and the use of only adult *L*. *decemlineata* in our study may explain this discrepancy. Additionally, although Trinity uses a complex set of equations to try to generate full length transcripts from contings, there may be fragmentation of genes with low coverage, including the cytochrome p450 family if genes are weakly expressed. The closest genome that has been annotated to *L*. *decemlineata* is *Tribolium castaneum*, and its genome revealed 132 Cyp genes [13] which is more similar in number to our annotation.

We also observed differences in gene expression between two second generation *L*. *decemlineata* populations through analysis of gene expression from whole *L*. *decemlineata* samples. We focused our study on previously described mechanisms of resistance, including phase one metabolism (cytochrome p450s) and phase two metabolism (glutathione S–transferases), as well as the metabolic signature of reduced penetrance and increased excretion. Previous studies have demonstrated the importance of cytochrome p450 in imidacloprid resistant populations of insects, including the overexpression of Cyp6g1 in *Drosophila melanogaster* [27] and Cyp6CM1 in *Bemisia tabaci* [28]. Another study demonstrated that cytochrome p450 inhibition in *L*. *decemlineata* using piperonyl butoxide re-establishes a more susceptible population [8]. When we examined up-regulated genes in two resistant populations, we found many possible mechanisms of resistance that could play a role in detoxification of imidacloprid including multiple up-regulated cytochrome p450s.

Without an annotated genome, we classified genes based on their similarities to known reference sequences. Our study revealed that the systemic-1 population had 14 transcripts which could be classified among the 8 molecular mechanism classes investigated, and 6 of these were similar in homology to one of the reference protein sequences encompassing the cytochrome p450 family. In comparison, the systemic-3 population had 46 transcripts that could be classified in one of the 8 classes. We also found that the systemic-3 population expressed large numbers of proteins which are functionally relevant with cuticular proteins (23 out of the 46 up-regulated molecular mechanisms), while the systemic-1 population had no up-regulated cuticular proteins. The enrichment analysis between the whole transcriptome and the systemic populations also revealed that both populations overexpressed oxidoreductase activity and the systemic-3 population overexpressed monooxygenase activity. Finally, we examined whether there were genes that were up-regulated in both resistant populations. When we compared genes across resistant populations we found 8 shared transcripts that could code for some type of metabolic resistance, including 3 cytochrome p450s and a single glutathione synthetase related protein. We also examined if there were any trends in the shared, up-regulated transcripts that encoded for a known mechanism of resistance. The three cytochrome p450 genes were all from the Cyp 9 family and matched best to a cytochrome p450 from *T*. *castaneum*. The 3 ABC transporter proteins, were also compared to each other. Two belonged to Subfamily C, and all three transcripts that encoded for ABC transporter had a closest match to *T*. *castaneum*. Possible mutations in the nAChR between the susceptible and resistant populations were also examined, but were omitted from this study due to the low depth of coverage of RNA sequencing at the sites. The RNA sequencing data is also from pooled individuals, so conclusions about individual genotypes from this data regarding mutations in the nAChR could be misleading.

Our study suggests that different populations of *L*. *decemlineata* are most likely using a mixture of similar and dissimilar genes to combat pesticide insults. Despite the fact that the resistant populations of *L*. *decemlineata* were collected from similar geographic and agricultural regions of the state of Wisconsin, we speculate that field populations of *L*. *decemlineata* have adapted to the unique trends in pesticide application at each field location, leading to differences in up-regulated transcripts in each resistant population. Naturally, some of the genes could be similar because *L*. *decemlineata* host plants contain high levels of glycoalkaloids and *L*. *decemlineata* has adapted to become an effective pest. This speculation is further supported because it has been previously shown that most *L*. *decemlineata* do not migrate more than 400m from their overwintering range in a given year [29]. Although some population mixing can certainly occur over agricultural landscapes, these observations further suggest that *L*. *decemlineata* genetics might be discrete in their distribution.

## Conclusions

The purpose of this study was to uncover potential genes responsible for imidacloprid resistance in spatially explicit *L*. *decemlineata* populations in the Central Sands region of Wisconsin. We used knowledge acquired from Huseth and Groves [4], together with dose-response generated regression estimates that we calculated in 2013 and 2014, to identify 2 candidate imidacloprid resistant field populations and a single susceptible population. With this data, we attempted to establish the mode of imidacloprid resistance within these selected populations of *L*. *decemlineata* by uncovering genes that were differentially expressed in association with imidacloprid exposure. We found that both of the resistant populations investigated had many up-regulated genes that were constitutively activated, including many cytochrome p450s. Interestingly cuticular proteins were found up-regulated in only the systemic-3 population. One limitation of this study is that it did not integrate the effects that other pesticides and environmental stressors have had on beetles. It is possible that some of the genes that are being up-regulated are the product of other stressors. We chose to use field populations of *L*. *decemlineata* to examine relevant field conditions that give rise to insecticide resistance that farmers face.

This study also aimed to better understand the beetle’s genome by creating a transcriptome from extracted RNA. We hypothesized that resistant populations would have differentially regulated gene expression when compared to susceptible populations. RNA sequencing and assembly technology provided us a comprehensive picture of genes that are expressed differently between selected populations of *L*. *decemlineata* in this region of Wisconsin. Even though populations of beetles are collected from fields within the same geographical region, the modes of action for insecticide resistance seem to vary in space, as multiple up-regulated genes vary among populations.

In particular, we demonstrated that multiple genes, or modes of action that could encode for pesticide resistance, are up-regulated in each of the two resistant populations. Studies have suggested that cytochrome p450 may play a role in the detoxification of imidacloprid and indeed we found evidence that these genes are up-regulated in resistant Wisconsin populations. The information provided from this study clarifies possible resistance mechanisms and can assist us in future efforts to determine good candidate genes for gene targeting pesticides (i.e. RNAi technology), such as the cytochrome p450 and cuticular proteins.

## Supporting Information

S1 Table Up-regulated components in the Systemic-1 population determined by a fold change of greater than 2 and a FDR of less than 0.059. (DOCX)

S2 Table Up-regulated components in the Systemic-3 population determined by a fold change of greater than 2 and a FDR of less than 0.059. (DOCX)

S3 Table Enrichment analysis between GO terms from the up-regulated transcripts of the systemic-1 population compared to the whole transcriptome. (DOCX)

S4 Table Enrichment analysis between GO terms from the up-regulated transcripts of the systemic-3 population compared to the whole transcriptome. (DOCX)
