# Supplementary material for: Characterizing Molecular Mechanisms of Imidacloprid Resistance in Select Populations of Leptinotarsa decemlineata in the Central Sands Region of Wisconsin
Source: PLoS One. 2016 Jan 28;11(1):e0147844. doi: 10.1371/journal.pone.0147844 (PMC4731083; doi:10.1371/journal.pone.0147844)
Supplement: S1 Table — (DOCX) [file pone.0147844.s001.docx]

**Supplementary Table S1:** Up-regulated components in the Systemic-1 population determined by a fold change of greater than 2 and a FDR of less than 0.059. Component Description is the reference gene associated with the blast hit from the reference database.

| Components | FDR | Fold Change | Component Description |
| --- | --- | --- | --- |
| comp100892_c0 | 0.001 | 9.730 | ---NA--- |
| comp100923_c0 | 0.001 | 2.674 | PREDICTED: similar to alpha-esterase |
| comp101093_c0 | 0.006 | 11.940 | ---NA--- |
| comp101549_c0 | 0.005 | 7.215 | caseinolytic peptidase b protein homolog (suppressor of potassium transport defect 3) |
| comp101816_c0 | 0.001 | 2.364 | juvenile hormone-inducible |
| comp101841_c0 | 0.040 | 2.525 | ---NA--- |
| comp101855_c0 | 0.054 | 3.017 | ---NA--- |
| comp101939_c0 | 0.001 | 2.443 | ---NA--- |
| comp102061_c0 | 0.001 | 3.174 | cathepsin b-like like proteinase |
| comp102186_c0 | 0.001 | 2.629 | AGAP009328-PA |
| comp102187_c1 | 0.026 | 2.241 | Actin, muscle |
| comp102265_c1 | 0.017 | 2.474 | ---NA--- |
| comp102305_c0 | 0.041 | 2.263 | cg9090 cg9090-pa |
| comp102424_c0 | 0.001 | 2.422 | translocator protein-like |
| comp102425_c0 | 0.001 | 3.388 | ---NA--- |
| comp102476_c0 | 0.001 | 21.655 | ---NA--- |
| comp102528_c0 | 0.041 | 2.013 | ---NA--- |
| comp102657_c0 | 0.011 | 2.389 | PREDICTED: similar to AGAP006424-PA |
| comp102690_c0 | 0.001 | 4.905 | PREDICTED: hypothetical protein |
| comp102761_c0 | 0.020 | 2.457 | 28 kda desiccation stress protein |
| comp102762_c0 | 0.003 | 2.846 | ---NA--- |
| comp102853_c0 | 0.001 | 2.304 | PREDICTED: similar to 3-oxoacyl- |
| comp102935_c0 | 0.001 | 7.199 | ---NA--- |
| comp103119_c0 | 0.001 | 3.230 | sodium-dependent phosphate transporter |
| comp103228_c0 | 0.053 | 2.091 | PREDICTED: hypothetical protein LOC100570164 |
| comp103236_c0 | 0.008 | 2.487 | AGAP007368-PA |
| comp103555_c0 | 0.001 | 4.097 | ---NA--- |
| comp103556_c0 | 0.057 | 4.272 | cg9427 cg9427-pa |
| comp103650_c0 | 0.001 | 13.195 | defensin 1 |
| comp103658_c0 | 0.001 | 2.373 | cytochrome p450 9z4 |
| comp103760_c0 | 0.001 | 3.113 | beta- -glucan recognition protein 2 |
| comp103910_c0 | 0.001 | 3.282 | ---NA--- |
| comp103967_c0 | 0.001 | 2.168 | PREDICTED: similar to AGAP001449-PA |
| comp104160_c0 | 0.015 | 2.940 | ---NA--- |
| comp104308_c0 | 0.002 | 2.207 | PREDICTED: similar to GA15997-PA |
| comp104384_c0 | 0.001 | 2.369 | ---NA--- |
| comp104411_c0 | 0.001 | 2.528 | 29-kda galactose-binding lectin |
| comp104533_c0 | 0.001 | 7.084 | acid phosphatase 1 |
| comp104646_c0 | 0.001 | 2.164 | defensin 1 |
| comp104702_c0 | 0.020 | 2.398 | ---NA--- |
| comp104711_c0 | 0.022 | 2.206 | cg6870 cg6870-pa |
| comp104713_c0 | 0.034 | 2.197 | niemann-pick type c-1b |
| comp104806_c0 | 0.001 | 2.003 | PREDICTED: similar to conserved hypothetical protein |
| comp104861_c0 | 0.003 | 2.326 | ---NA--- |
| comp104933_c0 | 0.001 | 3.334 | glucose dehydrogenase |
| comp104970_c0 | 0.001 | 2.164 | ---NA--- |
| comp104984_c0 | 0.001 | 8.539 | allergen bla g |
| comp105039_c1 | 0.032 | 2.567 | cathepsin l-like protein cysteine proteinase |
| comp105223_c0 | 0.001 | 2.291 | serine protease 2 |
| comp105323_c0 | 0.019 | 2.118 | ---NA--- |
| comp105434_c0 | 0.001 | 2.130 | juvenile hormone-inducible |
| comp105457_c0 | 0.058 | 2.066 | ---NA--- |
| comp105517_c0 | 0.051 | 2.057 | ---NA--- |
| comp105537_c2 | 0.001 | 3.163 | ---NA--- |
| comp105581_c0 | 0.037 | 2.038 | purine biosynthesis protein pur6 isoform 1 |
| comp105731_c1 | 0.001 | 2.325 | cg7675 cg7675-pb |
| comp105917_c0 | 0.005 | 3.144 | ---NA--- |
| comp105956_c0 | 0.013 | 2.012 | ---NA--- |
| comp105973_c0 | 0.001 | 3.302 | PREDICTED: similar to AGAP005972-PA |
| comp105989_c0 | 0.001 | 2.420 | AGAP011167-PA |
| comp106035_c1 | 0.001 | 6.524 | tryptophan -dioxygenase |
| comp106072_c0 | 0.001 | 2.504 | cytochrome p450 9z4 |
| comp106118_c0 | 0.003 | 2.143 | PREDICTED: similar to conserved hypothetical protein |
| comp106220_c0 | 0.001 | 2.867 | cg6870 cg6870-pa |
| comp106244_c0 | 0.001 | 4.736 | 12 kda hemolymph protein b |
| comp106294_c2 | 0.001 | 2.061 | atp-binding cassette transporter |
| comp106296_c0 | 0.015 | 2.594 | ---NA--- |
| comp106421_c0 | 0.026 | 2.346 | AGAP011167-PA |
| comp106543_c1 | 0.001 | 2.747 | poils au dos |
| comp106548_c0 | 0.001 | 2.517 | xanthine dehydrogenase |
| comp106668_c0 | 0.001 | 2.061 | cg6084 cg6084-pa |
| comp106677_c0 | 0.009 | 2.567 | AGAP011630-PA |
| comp106711_c0 | 0.001 | 2.163 | acid phosphatase-1 |
| comp106832_c0 | 0.006 | 2.289 | PREDICTED: similar to AGAP001553-PA |
| comp106834_c0 | 0.001 | 2.182 | AGAP003206-PB |
| comp107125_c0 | 0.001 | 2.385 | conserved hypothetical protein |
| comp107157_c0 | 0.021 | 2.248 | pacifastin-related serine protease inhibitor isoform 2 |
| comp107172_c0 | 0.001 | 2.076 | ---NA--- |
| comp107182_c0 | 0.001 | 2.169 | zinc-containing alcohol dehydrogenase |
| comp107277_c0 | 0.019 | 2.027 | variable lymphocyte receptor a |
| comp107290_c0 | 0.001 | 3.318 | PREDICTED: hypothetical protein LOC100165870 |
| comp107322_c0 | 0.001 | 2.375 | hemoglobin c1 polymer |
| comp107451_c0 | 0.020 | 2.460 | ---NA--- |
| comp107507_c0 | 0.001 | 2.123 | cg1673 cg1673-pa |
| comp107523_c0 | 0.030 | 2.675 | hypothetical protein Phum_PHXM454910 |
| comp107656_c0 | 0.001 | 3.503 | ---NA--- |
| comp107703_c0 | 0.001 | 5.077 | cg34115 cg34115-pa |
| comp107706_c0 | 0.001 | 4.201 | PREDICTED: similar to AGAP005839-PA |
| comp107777_c0 | 0.025 | 2.291 | ---NA--- |
| comp107842_c0 | 0.001 | 2.050 | seven in absentia 1b |
| comp107865_c0 | 0.006 | 2.013 | aldo-keto reductase |
| comp107875_c0 | 0.018 | 3.357 | PREDICTED: similar to AGAP010241-PA |
| comp107926_c0 | 0.001 | 3.099 | ---NA--- |
| comp108155_c0 | 0.001 | 5.841 | PREDICTED: hypothetical protein LOC100574147 |
| comp108241_c0 | 0.001 | 2.913 | PREDICTED: similar to AGAP003785-PA |
| comp108250_c0 | 0.001 | 2.027 | cg15105 cg15105-pa |
| comp108251_c0 | 0.001 | 5.033 | PREDICTED: hypothetical protein LOC100571634 |
| comp108262_c0 | 0.001 | 2.930 | 3-hydroxy-3-methylglutaryl- reductase |
| comp108539_c0 | 0.050 | 2.378 | cathepsin b-like cysteine proteinase-like |
| comp108541_c1 | 0.001 | 2.178 | ---NA--- |
| comp108561_c0 | 0.006 | 2.484 | cg3108 cg3108-pa |
| comp108640_c0 | 0.001 | 2.469 | ---NA--- |
| comp108695_c0 | 0.001 | 3.965 | ---NA--- |
| comp108705_c0 | 0.020 | 2.044 | Xracil-DNA glycosylase, putative |
| comp108777_c0 | 0.001 | 2.634 | acid phosphatase-1 |
| comp108826_c0 | 0.006 | 2.470 | peritrophic matrix protein 3 precursor |
| comp108902_c0 | 0.001 | 2.389 | PREDICTED: similar to AGAP001894-PA |
| comp108962_c1 | 0.003 | 2.358 | ---NA--- |
| comp109050_c0 | 0.001 | 3.063 | ---NA--- |
| comp109194_c0 | 0.020 | 2.015 | isoform a |
| comp109331_c0 | 0.010 | 2.519 | ---NA--- |
| comp109356_c0 | 0.013 | 2.340 | eukaryotic translation initiation factor 4e binding protein |
| comp109435_c0 | 0.001 | 2.349 | 29-kda galactose-binding lectin |
| comp109504_c1 | 0.001 | 2.412 | chitinase 3 precursor |
| comp109529_c0 | 0.001 | 2.328 | sodium-dependent phosphate transporter |
| comp109623_c0 | 0.001 | 14.366 | PREDICTED: hypothetical protein |
| comp109630_c0 | 0.001 | 2.468 | PREDICTED: hypothetical protein |
| comp109633_c0 | 0.005 | 2.203 | PREDICTED: similar to putative esterase |
| comp109639_c0 | 0.001 | 2.486 | sodium-dependent phosphate transporter |
| comp109647_c0 | 0.001 | 2.064 | PREDICTED: similar to conserved hypothetical protein |
| comp109680_c0 | 0.020 | 2.918 | juvenile hormone-inducible |
| comp109791_c1 | 0.001 | 2.495 | uncharacterized protein LOC662961 |
| comp109815_c1 | 0.033 | 2.313 | NA |
| comp109849_c0 | 0.001 | 2.018 | cg14275 cg14275-pa |
| comp109878_c0 | 0.001 | 3.644 | phosphodiesterase 9 cg32648-pa |
| comp109936_c0 | 0.001 | 2.954 | NA |
| comp109941_c0 | 0.010 | 2.597 | ---NA--- |
| comp110160_c0 | 0.002 | 2.068 | cg17664 cg17664-pb |
| comp110161_c0 | 0.001 | 3.175 | antennal-enriched xdp-glycosyltransferase |
| comp110172_c0 | 0.012 | 2.056 | ---NA--- |
| comp110251_c0 | 0.001 | 2.129 | cg9119 cg9119-pa |
| comp110258_c0 | 0.039 | 2.370 | cg6084 cg6084-pa |
| comp110286_c1 | 0.011 | 2.380 | equilibrative nucleoside |
| comp110308_c0 | 0.001 | 2.375 | PREDICTED: hypothetical protein LOC100570299 |
| comp110336_c0 | 0.002 | 2.103 | PREDICTED: similar to AGAP002198-PA |
| comp110381_c0 | 0.001 | 7.377 | ---NA--- |
| comp110446_c0 | 0.003 | 3.330 | peptidoglycan recognition protein short class (agap006343-pa) |
| comp110599_c0 | 0.002 | 2.048 | propionyl- carboxylase alpha mitochondrial precursor (pccase subunit alpha) (propanoyl- :carbon dioxide ligase subunit alpha) |
| comp110691_c0 | 0.024 | 2.247 | PREDICTED: similar to GA11424-PA |
| comp110698_c2 | 0.001 | 2.174 | ---NA--- |
| comp110718_c0 | 0.001 | 5.725 | alcohol dehydrogenase |
| comp110734_c0 | 0.001 | 2.713 | alcohol dehydrogenase |
| comp110813_c0 | 0.005 | 2.193 | glucosyl glucuronosyl transferases |
| comp110885_c0 | 0.001 | 2.620 | amino acid transporter |
| comp110906_c0 | 0.001 | 23.437 | PREDICTED: hypothetical protein |
| comp110935_c0 | 0.001 | 3.993 | pancreatic lipase |
| comp110977_c0 | 0.018 | 2.132 | lysosomal thiol reductase ip30 precursor |
| comp110997_c1 | 0.001 | 2.505 | cg3106 cg3106-pa |
| comp111069_c0 | 0.001 | 2.372 | peroxidasin homolog |
| comp111132_c0 | 0.001 | 2.439 | PREDICTED: similar to GA12046-PA |
| comp111231_c1 | 0.001 | 2.148 | ---NA--- |
| comp111255_c0 | 0.007 | 2.465 | ---NA--- |
| comp111326_c0 | 0.002 | 2.239 | transposable element p transposase (p-element transposase) |
| comp111372_c0 | 0.001 | 2.447 | PREDICTED: similar to AGAP002559-PA |
| comp111513_c0 | 0.038 | 2.254 | clip domain serine protease |
| comp111616_c0 | 0.001 | 2.759 | PREDICTED: similar to predicted protein |
| comp111631_c0 | 0.001 | 2.380 | ---NA--- |
| comp111641_c0 | 0.006 | 3.581 | AGAP002387-PA |
| comp111653_c0 | 0.001 | 3.354 | PREDICTED: similar to GA13362-PA |
| comp111660_c0 | 0.004 | 2.378 | ---NA--- |
| comp111665_c0 | 0.006 | 2.088 | AGAP002799-PA |
| comp111667_c1 | 0.001 | 2.802 | cg1213 cg1213-pa |
| comp111672_c0 | 0.001 | 2.971 | PREDICTED: similar to AGAP012156-PA |
| comp111691_c1 | 0.001 | 2.775 | cytochrome p450 monooxygenase |
| comp111701_c1 | 0.003 | 2.055 | cg5044 cg5044-pa |
| comp111719_c0 | 0.001 | 2.280 | cationic amino acid transporter |
| comp111768_c1 | 0.001 | 2.298 | juvenile hormone-inducible |
| comp111850_c0 | 0.001 | 2.931 | isoform a |
| comp111874_c0 | 0.001 | 2.042 | beta 1-like 2 |
| comp111910_c0 | 0.025 | 2.027 | PREDICTED: similar to AGAP006427-PA |
| comp111916_c1 | 0.051 | 2.048 | uncharacterized protein LOC662961 |
| comp111937_c0 | 0.001 | 3.705 | ---NA--- |
| comp111971_c0 | 0.001 | 2.037 | PREDICTED: similar to AGAP001553-PA |
| comp111980_c0 | 0.011 | 2.095 | PREDICTED: hypothetical protein LOC100570299 |
| comp111987_c1 | 0.001 | 2.145 | dimeric dihydrodiol dehydrogenase isoform 1 |
| comp112067_c1 | 0.001 | 2.100 | cytochrome p450 monooxygenase |
| comp112077_c0 | 0.001 | 2.031 | isoform a |
| comp112104_c0 | 0.001 | 2.261 | ---NA--- |
| comp112120_c0 | 0.002 | 2.009 | ---NA--- |
| comp112148_c0 | 0.001 | 3.030 | PREDICTED: similar to cystathionine-beta-synthase |
| comp112200_c0 | 0.018 | 2.113 | ---NA--- |
| comp112222_c0 | 0.010 | 2.145 | juvenile hormone-inducible |
| comp112294_c0 | 0.001 | 4.511 | ---NA--- |
| comp112295_c0 | 0.001 | 2.069 | tpr repeat-containing protein c9orf52 |
| comp112376_c0 | 0.002 | 2.017 | PREDICTED: hypothetical protein LOC100573156 |
| comp112427_c1 | 0.001 | 2.031 | PREDICTED: similar to F28G4.5 |
| comp112459_c0 | 0.001 | 2.906 | ventral nervous system defective |
| comp112482_c0 | 0.005 | 2.831 | ---NA--- |
| comp112570_c0 | 0.001 | 2.138 | PREDICTED: similar to chrysoptin |
| comp112604_c0 | 0.001 | 3.249 | PREDICTED: similar to GA19585-PA |
| comp112608_c0 | 0.001 | 2.056 | antennal-enriched xdp-glycosyltransferase |
| comp112638_c0 | 0.001 | 3.741 | ---NA--- |
| comp112664_c0 | 0.005 | 2.489 | ---NA--- |
| comp112685_c0 | 0.001 | 6.499 | AGAP005332-PC |
| comp112752_c0 | 0.001 | 2.116 | PREDICTED: similar to AGAP006569-PA |
| comp112759_c0 | 0.001 | 2.492 | PREDICTED: similar to AGAP000973-PA |
| comp112816_c0 | 0.001 | 5.786 | antennae-rich cytochrome p450 |
| comp112968_c0 | 0.001 | 2.625 | glucosyl glucuronosyl transferases |
| comp113028_c1 | 0.001 | 2.176 | aldo-keto reductase |
| comp113119_c1 | 0.002 | 2.181 | PREDICTED: similar to chrysoptin |
| comp113204_c0 | 0.009 | 2.061 | steroid dehydrogenase isoform 1 |
| comp113238_c1 | 0.001 | 2.352 | cathepsin b |
| comp113270_c0 | 0.001 | 2.655 | AGAP007074-PA |
| comp113283_c0 | 0.005 | 2.058 | PREDICTED: hypothetical protein LOC100573212, partial |
| comp113360_c0 | 0.001 | 3.071 | equilibrative nucleoside transporter 1 cg11907-pa |
| comp113424_c0 | 0.001 | 2.794 | PREDICTED: hypothetical protein LOC100575395 |
| comp113436_c1 | 0.001 | 2.040 | PREDICTED: hypothetical protein LOC100570299 |
| comp113475_c0 | 0.001 | 2.435 | PREDICTED: hypothetical protein |
| comp113507_c0 | 0.003 | 2.046 | lipase 3 |
| comp113542_c1 | 0.001 | 2.676 | scavenger receptor acting in neural tissue and majority of rhodopsin is absent cg12789-pb |
| comp113584_c0 | 0.002 | 2.057 | PREDICTED: similar to GA18316-PA |
| comp113592_c0 | 0.001 | 2.974 | lysosomal acid lipase |
| comp113607_c1 | 0.001 | 2.034 | inebriated protein |
| comp113625_c1 | 0.001 | 3.771 | bifunctional protein fold |
| comp113636_c0 | 0.001 | 3.098 | glucose dehydrogenase |
| comp113703_c0 | 0.001 | 2.560 | ---NA--- |
| comp113704_c0 | 0.001 | 2.140 | beta 1-like 2 |
| comp113764_c0 | 0.001 | 2.434 | sodium-dependent phosphate transporter |
| comp113913_c0 | 0.001 | 2.302 | ankyrin repeat |
| comp113948_c0 | 0.001 | 6.193 | PREDICTED: similar to Luciferase |
| comp113982_c0 | 0.005 | 2.046 | PREDICTED: hypothetical protein LOC100569635 |
| comp114026_c0 | 0.001 | 2.089 | glutathione synthetase |
| comp114049_c1 | 0.001 | 2.526 | ---NA--- |
| comp114076_c0 | 0.001 | 2.530 | ornithine decarboxylase |
| comp114081_c0 | 0.001 | 3.623 | PREDICTED: similar to alpha-esterase |
| comp114139_c0 | 0.012 | 2.192 | ---NA--- |
| comp114166_c0 | 0.005 | 2.234 | PREDICTED: similar to AGAP008487-PA |
| comp114174_c0 | 0.001 | 2.211 | PREDICTED: similar to 4-nitrophenylphosphatase |
| comp114295_c0 | 0.001 | 2.132 | cgmp-dependent protein kinase |
| comp114343_c0 | 0.056 | 2.079 | PREDICTED: similar to carboxylesterase |
| comp114363_c0 | 0.004 | 2.772 | ---NA--- |
| comp114539_c1 | 0.055 | 2.171 | ---NA--- |
| comp114570_c0 | 0.001 | 2.381 | cg12340 cg12340-pa |
| comp114596_c0 | 0.020 | 3.412 | PREDICTED: similar to AGAP002559-PA |
| comp114629_c0 | 0.012 | 2.416 | histone-lysine n-methyltransferase setmar-like |
| comp114643_c0 | 0.001 | 2.891 | PREDICTED: similar to AGAP009114-PA |
| comp114658_c0 | 0.001 | 7.200 | PREDICTED: hypothetical protein |
| comp114838_c0 | 0.001 | 2.406 | cad88c cg3389-pa |
| comp114870_c0 | 0.001 | 3.529 | PREDICTED: similar to beta-glucosidase |
| comp114950_c0 | 0.052 | 2.003 | ---NA--- |
| comp114996_c1 | 0.001 | 2.064 | cg4382 cg4382-pa |
| comp115030_c0 | 0.001 | 3.015 | ---NA--- |
| comp115076_c0 | 0.001 | 2.401 | PREDICTED: hypothetical protein |
| comp115119_c0 | 0.001 | 2.290 | ---NA--- |
| comp115141_c0 | 0.015 | 2.300 | PREDICTED: similar to AGAP012043-PA |
| comp115178_c0 | 0.001 | 5.170 | sodium solute symporter |
| comp115194_c0 | 0.001 | 4.482 | sodium solute symporter |
| comp115223_c0 | 0.015 | 2.238 | ---NA--- |
| comp115261_c0 | 0.001 | 2.075 | e3 ubiquitin-protein ligase siah2 (seven in absentia homolog 2-like) (siah-2) |
| comp115479_c0 | 0.001 | 2.682 | ---NA--- |
| comp115507_c0 | 0.001 | 3.920 | PREDICTED: similar to conserved hypothetical protein |
| comp115654_c0 | 0.001 | 2.512 | matrix metalloproteinase |
| comp115666_c0 | 0.003 | 2.328 | farnesyl pyrophosphate synthase |
| comp115689_c0 | 0.001 | 2.472 | sodium solute symporter |
| comp115735_c0 | 0.001 | 3.236 | kaz1-orfb cg1220-pe |
| comp115775_c0 | 0.001 | 2.250 | sodium nucleoside cotransporter |
| comp115842_c1 | 0.001 | 2.481 | chitinase 2 precursor |
| comp115848_c0 | 0.001 | 2.882 | sodium-dependent phosphate transporter |
| comp115889_c0 | 0.053 | 2.334 | rna-directed dna polymerase from mobile element jockey-like |
| comp115891_c0 | 0.005 | 2.002 | ---NA--- |
| comp115908_c0 | 0.001 | 2.165 | PREDICTED: similar to C25F9.2, partial |
| comp115954_c0 | 0.001 | 2.277 | antennal-enriched xdp-glycosyltransferase |
| comp115994_c0 | 0.053 | 2.609 | PREDICTED: similar to AGAP004918-PA |
| comp116046_c1 | 0.001 | 2.926 | equilibrative nucleoside transporter 1 cg11907-pa |
| comp116113_c0 | 0.001 | 2.397 | PREDICTED: similar to AGAP003785-PA |
| comp116205_c0 | 0.001 | 2.036 | ankyrin unc44 |
| comp116246_c0 | 0.001 | 2.020 | PREDICTED: similar to GA10859-PA |
| comp116262_c0 | 0.001 | 3.195 | cg1213 cg1213-pa |
| comp116489_c0 | 0.001 | 2.052 | chitin synthase 2 |
| comp116495_c1 | 0.001 | 2.086 | sid-1-related a precursor |
| comp116505_c0 | 0.001 | 2.069 | ---NA--- |
| comp116539_c1 | 0.001 | 2.310 | probable galactose-1-phosphate uridylyltransferase-like |
| comp116612_c0 | 0.018 | 2.058 | ---NA--- |
| comp116616_c0 | 0.007 | 2.034 | PREDICTED: similar to AGAP012154-PA |
| comp116671_c0 | 0.001 | 2.251 | ---NA--- |
| comp116743_c1 | 0.001 | 2.284 | lysosomal alpha-mannosidase (mannosidase alpha class 2b member 1) |
| comp116803_c0 | 0.001 | 3.867 | PREDICTED: similar to AGAP008487-PA |
| comp116806_c2 | 0.015 | 2.054 | membrane alanyl aminopeptidase |
| comp116830_c1 | 0.030 | 2.361 | ---NA--- |
| comp116900_c0 | 0.001 | 2.120 | cgmp-dependent protein kinase |
| comp116927_c1 | 0.038 | 3.190 | ---NA--- |
| comp116958_c0 | 0.001 | 3.200 | labial |
| comp116970_c0 | 0.016 | 2.002 | forked cg5424-pb |
| comp117027_c0 | 0.007 | 2.044 | cg8709 cg8709-pb |
| comp117070_c0 | 0.001 | 2.877 | transposable element p transposase (p-element transposase) |
| comp117074_c0 | 0.001 | 2.526 | diphosphomevalonate decarboxylase |
| comp117076_c0 | 0.001 | 9.178 | glucose dehydrogenase |
| comp117132_c0 | 0.001 | 2.729 | PREDICTED: similar to phosphoribosylformylglycinamidine synthase, putative |
| comp117214_c0 | 0.001 | 2.673 | reverse transcriptase homolog |
| comp117241_c0 | 0.006 | 2.305 | ras-like gtp-binding protein rho1 |
| comp117263_c0 | 0.050 | 2.208 | ---NA--- |
| comp117365_c0 | 0.001 | 2.008 | npc1 protein |
| comp117371_c0 | 0.001 | 2.433 | multi drug resistance 50 cg8523-pa |
| comp117423_c1 | 0.001 | 2.479 | amp dependent ligase |
| comp117438_c0 | 0.023 | 2.088 | isoform a |
| comp117469_c0 | 0.001 | 2.356 | sugar transporter |
| comp117494_c0 | 0.046 | 2.177 | laccase 1 |
| comp117534_c0 | 0.001 | 2.082 | ---NA--- |
| comp117547_c0 | 0.001 | 2.057 | PREDICTED: similar to conserved hypothetical protein |
| comp117554_c0 | 0.027 | 2.037 | fatty acid synthase-like |
| comp117597_c0 | 0.001 | 3.503 | elongation of very long chain fatty acids protein aael008004-like |
| comp117622_c0 | 0.001 | 2.257 | cg7044 cg7044-pa |
| comp117771_c0 | 0.001 | 7.446 | ---NA--- |
| comp117805_c0 | 0.001 | 3.615 | ---NA--- |
| comp117812_c0 | 0.034 | 2.272 | kynurenine alpha-aminoadipate aminotransferase mitochondrial precursor (kat ) (kynurenine--oxoglutarate transaminase ii) (kynurenine aminotransferase ii) (kynurenine--oxoglutarate aminotransferase ii) (2-aminoadipate transaminase) ( |
| comp117821_c0 | 0.001 | 2.068 | atp-binding sub-family c (cftr mrp) member 4 |
| comp117866_c0 | 0.001 | 2.046 | PREDICTED: similar to AGAP006427-PA |
| comp117882_c0 | 0.001 | 2.695 | AGAP012173-PA |
| comp117934_c0 | 0.001 | 4.610 | xanthine dehydrogenase oxidase |
| comp118021_c0 | 0.001 | 2.119 | atp-binding cassette transporter |
| comp118072_c0 | 0.001 | 2.649 | xanthine dehydrogenase |
| comp118086_c0 | 0.001 | 2.762 | zinc finger bed domain-containing protein 5-like |
| comp118104_c1 | 0.001 | 3.141 | 3-hydroxy-3-methylglutaryl coenzyme a synthase |
| comp118166_c0 | 0.034 | 2.020 | cg41538 cg41538-pa |
| comp118239_c0 | 0.001 | 2.222 | tripsin, putative |
| comp118287_c1 | 0.012 | 2.221 | AGAP012074-PA |
| comp118327_c0 | 0.016 | 2.268 | agap008849-pa isoform 1 |
| comp118337_c0 | 0.035 | 2.481 | hemoglobin c1 polymer |
| comp118465_c0 | 0.001 | 2.435 | PREDICTED: similar to beta-glucosidase |
| comp118468_c0 | 0.001 | 2.200 | PREDICTED: similar to AGAP005839-PA |
| comp118479_c0 | 0.001 | 2.256 | xanthine dehydrogenase |
| comp118549_c0 | 0.002 | 2.165 | ---NA--- |
| comp118656_c0 | 0.001 | 2.076 | cg31116 cg31116-pe |
| comp118737_c0 | 0.021 | 2.392 | fatty acid |
| comp118743_c0 | 0.001 | 2.331 | fatty acid synthase |
| comp118800_c0 | 0.002 | 4.100 | agap002830-pa isoform 5 |
| comp118846_c0 | 0.010 | 2.360 | glycosyltransferase 25 family member |
| comp118914_c0 | 0.002 | 2.329 | PREDICTED: similar to polyprotein |
| comp118999_c0 | 0.001 | 2.762 | PREDICTED: similar to AGAP006427-PA |
| comp119092_c0 | 0.057 | 2.084 | oxidase peroxidase |
| comp119311_c1 | 0.001 | 2.099 | PREDICTED: similar to AGAP012156-PA |
| comp119434_c0 | 0.030 | 2.371 | PREDICTED: hypothetical protein LOC100573963 |
| comp119437_c0 | 0.002 | 2.052 | AGAP004533-PA |
| comp119500_c0 | 0.017 | 2.770 | ---NA--- |
| comp119526_c0 | 0.009 | 2.040 | muscle-specific protein 300 cg33715-pd |
| comp119545_c0 | 0.001 | 2.360 | glucosyl glucuronosyl transferases |
| comp119560_c1 | 0.030 | 2.051 | sugar transporter |
| comp119617_c1 | 0.032 | 2.053 | juvenile hormone-inducible |
| comp119690_c0 | 0.001 | 2.915 | ---NA--- |
| comp119777_c0 | 0.003 | 2.180 | cathepsin d isoform 1 |
| comp119857_c0 | 0.051 | 3.065 | xbiquitin-conjugating enzyme e2-17 kda (xbiquitin-protein ligase) (xbiquitin carrier protein) (protein effete) |
| comp119907_c0 | 0.016 | 3.255 | ---NA--- |
| comp119921_c0 | 0.007 | 5.425 | ---NA--- |
| comp119922_c0 | 0.031 | 3.110 | ---NA--- |
| comp119955_c0 | 0.013 | 4.895 | ---NA--- |
| comp120081_c0 | 0.032 | 4.448 | ---NA--- |
| comp120251_c0 | 0.031 | 2.400 | ---NA--- |
| comp46473_c0 | 0.046 | 2.375 | adenine nucleotide translocase isoform a |
| comp46590_c0 | 0.001 | 2.235 | ---NA--- |
| comp57556_c0 | 0.047 | 2.180 | ---NA--- |
| comp61890_c0 | 0.042 | 3.395 | AGAP000462-PA |
| comp62119_c0 | 0.059 | 2.690 | ---NA--- |
| comp62480_c0 | 0.006 | 5.220 | ---NA--- |
| comp63542_c0 | 0.032 | 3.640 | ---NA--- |
| comp63545_c0 | 0.001 | 3.190 | elongation factor isoform a |
| comp65111_c0 | 0.006 | 2.695 | PREDICTED: similar to putative esterase |
| comp65689_c0 | 0.043 | 2.240 | ---NA--- |
| comp66456_c0 | 0.006 | 2.845 | AGAP010885-PA |
| comp67283_c0 | 0.031 | 4.580 | AGAP009623-PA |
| comp68119_c0 | 0.058 | 2.100 | ---NA--- |
| comp68160_c0 | 0.034 | 2.930 | cg6084 cg6084-pa |
| comp79274_c0 | 0.010 | 3.955 | PREDICTED: similar to AGAP003584-PA |
| comp80300_c0 | 0.051 | 3.210 | ---NA--- |
| comp80468_c0 | 0.006 | 7.450 | tripartite motif-containing protein 2-like |
| comp81149_c0 | 0.028 | 2.860 | cytochrome p450 isoform 1 |
| comp82505_c0 | 0.041 | 2.020 | isoform a |
| comp82707_c0 | 0.008 | 4.660 | ---NA--- |
| comp85114_c0 | 0.048 | 3.030 | PREDICTED: hypothetical protein LOC100166108 isoform 3 |
| comp85230_c0 | 0.001 | 14.250 | ---NA--- |
| comp87864_c0 | 0.017 | 2.178 | calcium-transporting atpase type 2c |
| comp88149_c0 | 0.006 | 5.040 | ---NA--- |
| comp88335_c0 | 0.006 | 8.220 | small heat shock protein 21 isoform 1 |
| comp88374_c0 | 0.048 | 3.567 | ---NA--- |
| comp89182_c0 | 0.001 | 3.049 | ---NA--- |
| comp91024_c0 | 0.025 | 3.530 | ---NA--- |
| comp92340_c0 | 0.006 | 2.805 | 2-hydroxyphytanoyl-CoA lyase, putative |
| comp92480_c0 | 0.031 | 3.950 | superoxide dismutase 2 |
| comp93486_c0 | 0.009 | 3.695 | isoform e |
| comp93921_c0 | 0.006 | 6.235 | lethal essential for life l2efl |
| comp94687_c0 | 0.009 | 5.442 | ---NA--- |
| comp94693_c0 | 0.006 | 5.155 | Catalase, putative |
| comp95046_c0 | 0.006 | 3.810 | AGAP006958-PA |
| comp95711_c0 | 0.001 | 4.987 | ---NA--- |
| comp95983_c0 | 0.001 | 7.315 | PREDICTED: hypothetical protein |
| comp96315_c0 | 0.039 | 3.531 | AGAP002599-PA |
| comp96596_c0 | 0.050 | 2.425 | ---NA--- |
| comp97310_c0 | 0.002 | 6.450 | heat shock 70 kda protein |
| comp97809_c0 | 0.051 | 4.040 | ---NA--- |
| comp97937_c0 | 0.011 | 2.535 | AGAP004936-PA |
| comp98499_c0 | 0.059 | 2.045 | ---NA--- |
| comp99067_c0 | 0.001 | 3.568 | ---NA--- |
| comp99795_c0 | 0.023 | 2.030 | pro-phenol oxidase subunit 2 |
| comp99864_c0 | 0.058 | 2.162 | 13 kda hemolymph protein a |
| comp99927_c0 | 0.001 | 7.737 | ---NA--- |
