# Supplementary material for: Characterizing Molecular Mechanisms of Imidacloprid Resistance in Select Populations of Leptinotarsa decemlineata in the Central Sands Region of Wisconsin
Source: PLoS One. 2016 Jan 28;11(1):e0147844. doi: 10.1371/journal.pone.0147844 (PMC4731083; doi:10.1371/journal.pone.0147844)
Supplement: S2 Table — (DOCX) [file pone.0147844.s002.docx]

**Supplementary Table S2:** Up-regulated components in the Systemic-3 population determined by a fold change of greater than 2 and a FDR of less than 0.059. Component Description is the reference gene associated with the blast hit from the reference database.

| Components | FDR | Fold Change | Seq. Description |
| --- | --- | --- | --- |
| comp100068_c0 | 0.001 | 3.304 | ---NA--- |
| comp100083_c0 | 0.001 | 4.260 | CG32603 |
| comp100494_c0 | 0.001 | 4.684 | immune-related hdd11 |
| comp100635_c0 | 0.008 | 2.370 | PREDICTED: hypothetical protein |
| comp100776_c0 | 0.001 | 6.073 | ---NA--- |
| comp100892_c0 | 0.019 | 3.983 | ---NA--- |
| comp100923_c0 | 0.001 | 2.946 | PREDICTED: similar to alpha-esterase |
| comp101002_c0 | 0.057 | 2.070 | ---NA--- |
| comp101214_c0 | 0.001 | 3.252 | ---NA--- |
| comp101314_c0 | 0.001 | 2.580 | PREDICTED: similar to AGAP007667-PA |
| comp101651_c0 | 0.003 | 2.677 | ---NA--- |
| comp101692_c0 | 0.001 | 2.981 | pupal cuticle |
| comp101721_c0 | 0.001 | 4.930 | allergen aca s 13 |
| comp101742_c0 | 0.056 | 2.484 | ---NA--- |
| comp101816_c0 | 0.001 | 2.535 | juvenile hormone-inducible |
| comp101926_c0 | 0.005 | 2.542 | ---NA--- |
| comp101927_c0 | 0.001 | 2.466 | ---NA--- |
| comp101938_c0 | 0.002 | 2.447 | lysosomal thiol reductase ip30 precursor |
| comp101939_c0 | 0.001 | 2.309 | ---NA--- |
| comp101978_c0 | 0.001 | 4.332 | 25d cg6514-pa |
| comp101995_c0 | 0.001 | 4.813 | ---NA--- |
| comp102065_c0 | 0.034 | 2.235 | cg31760 cg31760-pa |
| comp102070_c0 | 0.039 | 2.543 | ---NA--- |
| comp102166_c0 | 0.001 | 3.314 | ---NA--- |
| comp102181_c0 | 0.001 | 4.701 | ---NA--- |
| comp102186_c0 | 0.025 | 2.151 | AGAP009328-PA |
| comp102265_c1 | 0.001 | 13.051 | ---NA--- |
| comp102272_c0 | 0.024 | 2.280 | AGAP011412-PA |
| comp102291_c0 | 0.001 | 8.210 | ---NA--- |
| comp102305_c0 | 0.007 | 2.606 | cg9090 cg9090-pa |
| comp102387_c0 | 0.035 | 2.125 | ---NA--- |
| comp102424_c0 | 0.001 | 3.294 | translocator protein-like |
| comp102425_c0 | 0.001 | 2.950 | ---NA--- |
| comp102476_c0 | 0.001 | 9.864 | ---NA--- |
| comp102545_c0 | 0.001 | 2.252 | PREDICTED: similar to conserved hypothetical protein |
| comp102636_c0 | 0.006 | 2.629 | pheromone binding protein |
| comp102657_c0 | 0.010 | 2.345 | PREDICTED: similar to AGAP006424-PA |
| comp102733_c0 | 0.010 | 2.092 | AGAP001409-PA |
| comp102743_c0 | 0.058 | 2.233 | ---NA--- |
| comp102762_c0 | 0.034 | 2.110 | ---NA--- |
| comp102853_c0 | 0.001 | 3.005 | PREDICTED: similar to 3-oxoacyl- |
| comp102935_c0 | 0.028 | 3.623 | ---NA--- |
| comp102949_c0 | 0.020 | 2.175 | cg34184 cg34184-pa |
| comp103172_c0 | 0.001 | 2.549 | ---NA--- |
| comp103236_c0 | 0.001 | 4.159 | AGAP007368-PA |
| comp103269_c0 | 0.001 | 2.437 | ---NA--- |
| comp103293_c0 | 0.033 | 2.363 | ---NA--- |
| comp103300_c0 | 0.043 | 2.522 | ---NA--- |
| comp103326_c0 | 0.001 | 5.995 | chemosensory protein 19 precursor |
| comp103377_c0 | 0.001 | 3.275 | chemosensory protein 1 precursor |
| comp103527_c0 | 0.010 | 2.271 | cral trio domain-containing protein |
| comp103555_c0 | 0.012 | 2.660 | ---NA--- |
| comp103556_c0 | 0.002 | 6.068 | cg9427 cg9427-pa |
| comp103564_c0 | 0.001 | 3.489 | cuticle protein cp5 |
| comp103650_c0 | 0.001 | 6.988 | defensin 1 |
| comp103658_c0 | 0.001 | 2.340 | cytochrome p450 9z4 |
| comp103662_c0 | 0.001 | 2.498 | AGAP010929-PA |
| comp103663_c0 | 0.001 | 2.588 | PREDICTED: similar to AGAP012342-PA |
| comp103671_c0 | 0.001 | 2.530 | PREDICTED: similar to alpha-esterase |
| comp103717_c0 | 0.001 | 6.594 | ---NA--- |
| comp103824_c0 | 0.040 | 2.810 | ---NA--- |
| comp103893_c0 | 0.003 | 2.250 | ---NA--- |
| comp103910_c0 | 0.001 | 14.484 | ---NA--- |
| comp103926_c0 | 0.001 | 3.171 | CG12947 |
| comp103954_c0 | 0.031 | 2.105 | AGAP011167-PA |
| comp103967_c0 | 0.001 | 2.086 | PREDICTED: similar to AGAP001449-PA |
| comp104036_c0 | 0.001 | 4.070 | ---NA--- |
| comp104056_c0 | 0.012 | 2.077 | methyltransferase 1 |
| comp104142_c0 | 0.004 | 2.033 | ---NA--- |
| comp104213_c0 | 0.001 | 2.641 | cg2837 cg2837-pd |
| comp104218_c0 | 0.001 | 2.445 | ---NA--- |
| comp104411_c0 | 0.002 | 2.400 | 29-kda galactose-binding lectin |
| comp104425_c0 | 0.001 | 2.790 | cytochrome p450 cyp9z1 |
| comp104506_c0 | 0.001 | 7.893 | ---NA--- |
| comp104517_c0 | 0.052 | 2.370 | antennal-enriched xdp-glycosyltransferase |
| comp104533_c0 | 0.001 | 4.664 | acid phosphatase 1 |
| comp104559_c0 | 0.007 | 3.595 | atp-dependent dna helicase pif1-like |
| comp104596_c1 | 0.024 | 3.143 | ---NA--- |
| comp104679_c0 | 0.003 | 2.523 | PREDICTED: hypothetical protein LOC100163076 |
| comp104714_c1 | 0.005 | 2.699 | ---NA--- |
| comp104719_c2 | 0.002 | 2.229 | ---NA--- |
| comp104806_c0 | 0.001 | 2.571 | PREDICTED: similar to conserved hypothetical protein |
| comp104861_c0 | 0.001 | 2.916 | ---NA--- |
| comp104933_c0 | 0.001 | 3.560 | glucose dehydrogenase |
| comp104984_c0 | 0.001 | 23.172 | allergen bla g |
| comp105013_c0 | 0.001 | 4.171 | ---NA--- |
| comp105039_c1 | 0.001 | 3.717 | cathepsin l-like protein cysteine proteinase |
| comp105058_c0 | 0.001 | 3.335 | ---NA--- |
| comp105087_c0 | 0.014 | 2.121 | protein takeout-like |
| comp105133_c0 | 0.010 | 2.041 | ---NA--- |
| comp105180_c0 | 0.017 | 2.230 | ---NA--- |
| comp105213_c0 | 0.006 | 2.551 | ---NA--- |
| comp105223_c0 | 0.001 | 2.292 | serine protease 2 |
| comp105250_c0 | 0.001 | 3.505 | ---NA--- |
| comp105273_c0 | 0.001 | 2.981 | cg9427 cg9427-pa |
| comp105274_c0 | 0.001 | 10.018 | cg30101 cg30101-pa |
| comp105406_c0 | 0.007 | 2.245 | PREDICTED: similar to AGAP011167-PA |
| comp105412_c0 | 0.001 | 3.775 | AGAP000697-PB |
| comp105434_c0 | 0.001 | 2.240 | juvenile hormone-inducible |
| comp105537_c2 | 0.001 | 2.333 | ---NA--- |
| comp105555_c0 | 0.051 | 2.301 | PREDICTED: hypothetical protein LOC100575767 |
| comp105589_c0 | 0.001 | 4.511 | resilin precursor |
| comp105656_c0 | 0.001 | 3.109 | protein takeout |
| comp105686_c0 | 0.003 | 2.085 | sodium-dependent phosphate transporter |
| comp105691_c0 | 0.035 | 2.262 | oxidase peroxidase |
| comp105731_c1 | 0.001 | 2.675 | cg7675 cg7675-pb |
| comp105797_c1 | 0.045 | 2.367 | ---NA--- |
| comp105859_c0 | 0.002 | 2.812 | AGAP007368-PA |
| comp105889_c0 | 0.001 | 6.484 | cuticular protein 92f cg5494-pa |
| comp105908_c0 | 0.025 | 2.014 | ---NA--- |
| comp105972_c0 | 0.026 | 2.450 | ---NA--- |
| comp105989_c0 | 0.001 | 3.066 | AGAP011167-PA |
| comp106035_c1 | 0.001 | 8.811 | tryptophan -dioxygenase |
| comp106072_c0 | 0.001 | 2.237 | cytochrome p450 9z4 |
| comp106188_c0 | 0.001 | 4.339 | cytochrome p450 cyp18a1 |
| comp106193_c0 | 0.001 | 2.979 | PREDICTED: similar to AGAP002559-PA |
| comp106220_c0 | 0.001 | 10.482 | cg6870 cg6870-pa |
| comp106242_c0 | 0.001 | 2.431 | PREDICTED: similar to AGAP001552-PA |
| comp106244_c0 | 0.001 | 5.616 | 12 kda hemolymph protein b |
| comp106245_c0 | 0.059 | 2.682 | valyl-trna synthetase |
| comp106259_c0 | 0.021 | 3.186 | ---NA--- |
| comp106283_c0 | 0.001 | 2.066 | CG17571 |
| comp106293_c0 | 0.002 | 2.055 | PREDICTED: similar to conserved hypothetical protein |
| comp106395_c0 | 0.003 | 2.431 | uncharacterized protein LOC662961 |
| comp106483_c0 | 0.001 | 4.137 | ---NA--- |
| comp106539_c0 | 0.001 | 2.639 | anterior fat body protein |
| comp106543_c1 | 0.001 | 2.413 | poils au dos |
| comp106548_c0 | 0.001 | 2.771 | xanthine dehydrogenase |
| comp106611_c0 | 0.001 | 3.663 | PREDICTED: similar to AGAP002557-PA |
| comp106668_c0 | 0.001 | 2.347 | cg6084 cg6084-pa |
| comp106677_c0 | 0.008 | 2.478 | AGAP011630-PA |
| comp106688_c2 | 0.040 | 2.146 | ---NA--- |
| comp106704_c0 | 0.001 | 2.342 | PREDICTED: similar to predicted protein |
| comp106740_c1 | 0.002 | 2.386 | ---NA--- |
| comp106758_c0 | 0.001 | 2.747 | cuticular protein rr-1 family (agap009876-pa) |
| comp106772_c1 | 0.001 | 4.530 | ---NA--- |
| comp106810_c0 | 0.001 | 4.594 | cg13024 cg13024-pa |
| comp106832_c0 | 0.001 | 2.782 | PREDICTED: similar to AGAP001553-PA |
| comp106834_c0 | 0.001 | 2.270 | AGAP003206-PB |
| comp106883_c0 | 0.001 | 2.360 | equilibrative nucleoside transporter 1 cg11907-pa |
| comp106947_c0 | 0.001 | 2.057 | ---NA--- |
| comp106953_c1 | 0.001 | 4.347 | ---NA--- |
| comp107026_c0 | 0.001 | 6.918 | ---NA--- |
| comp107049_c0 | 0.001 | 2.189 | chitin deacetylase 4 precursor |
| comp107059_c0 | 0.005 | 7.422 | ---NA--- |
| comp107072_c0 | 0.001 | 3.914 | ---NA--- |
| comp107080_c0 | 0.001 | 3.412 | ---NA--- |
| comp107106_c0 | 0.001 | 2.115 | cathepsin b precursor |
| comp107125_c0 | 0.001 | 2.312 | conserved hypothetical protein |
| comp107160_c0 | 0.023 | 2.543 | ---NA--- |
| comp107182_c0 | 0.001 | 3.087 | zinc-containing alcohol dehydrogenase |
| comp107230_c0 | 0.001 | 2.173 | ---NA--- |
| comp107286_c0 | 0.001 | 2.272 | ---NA--- |
| comp107290_c0 | 0.001 | 4.174 | PREDICTED: hypothetical protein LOC100165870 |
| comp107294_c0 | 0.001 | 2.089 | PREDICTED: hypothetical protein |
| comp107322_c0 | 0.001 | 2.387 | hemoglobin c1 polymer |
| comp107323_c0 | 0.008 | 2.566 | allergen aca s 13 |
| comp107326_c0 | 0.001 | 2.541 | ---NA--- |
| comp107348_c0 | 0.001 | 3.400 | glucose dehydrogenase |
| comp107354_c1 | 0.011 | 2.015 | ecdysteroid regulated 16 kda |
| comp107401_c0 | 0.026 | 2.456 | ---NA--- |
| comp107418_c0 | 0.001 | 5.426 | cuticle protein precursor |
| comp107435_c0 | 0.001 | 9.305 | PREDICTED: hypothetical protein |
| comp107462_c1 | 0.004 | 2.314 | ---NA--- |
| comp107494_c1 | 0.001 | 2.390 | retinol dehydrogenase 11 |
| comp107507_c0 | 0.001 | 2.397 | cg1673 cg1673-pa |
| comp107534_c2 | 0.001 | 2.193 | alcohol dehydrogenase |
| comp107589_c0 | 0.001 | 2.267 | transmembrane protein 195 |
| comp107656_c0 | 0.001 | 14.979 | ---NA--- |
| comp107703_c0 | 0.001 | 4.532 | cg34115 cg34115-pa |
| comp107706_c0 | 0.001 | 3.185 | PREDICTED: similar to AGAP005839-PA |
| comp107777_c0 | 0.017 | 2.317 | ---NA--- |
| comp107779_c0 | 0.001 | 8.553 | ---NA--- |
| comp107865_c0 | 0.001 | 2.268 | aldo-keto reductase |
| comp107899_c0 | 0.001 | 2.063 | ribosomal protein l36e |
| comp107917_c0 | 0.002 | 2.102 | PREDICTED: similar to AGAP008125-PA |
| comp107926_c0 | 0.001 | 3.397 | ---NA--- |
| comp107960_c0 | 0.001 | 3.519 | cg8927 cg8927-pa |
| comp107984_c0 | 0.017 | 2.383 | ---NA--- |
| comp108102_c0 | 0.001 | 5.555 | cytochrome p450 |
| comp108174_c0 | 0.022 | 2.057 | ---NA--- |
| comp108181_c0 | 0.019 | 2.037 | ---NA--- |
| comp108220_c1 | 0.001 | 2.423 | tyrosine-protein kinase |
| comp108225_c0 | 0.014 | 2.305 | conserved hypothetical protein |
| comp108241_c0 | 0.001 | 4.197 | PREDICTED: similar to AGAP003785-PA |
| comp108251_c0 | 0.001 | 15.79 | PREDICTED: hypothetical protein LOC100571634 |
| comp108257_c1 | 0.001 | 2.647 | luciferin-regenerating enzyme |
| comp108273_c1 | 0.001 | 2.035 | ---NA--- |
| comp108285_c0 | 0.001 | 2.280 | yellow-c precursor |
| comp108290_c1 | 0.001 | 2.127 | low density lipoprotein receptor adapter protein 1 (autosomal recessive hypercholesterolemia protein) isoform 1 |
| comp108325_c0 | 0.058 | 2.000 | ---NA--- |
| comp108333_c0 | 0.001 | 5.926 | cuticular protein 49aa cg30045-pb |
| comp108385_c0 | 0.001 | 2.342 | ---NA--- |
| comp108398_c0 | 0.013 | 2.426 | cuticular protein 47ef cg13214-pa |
| comp108416_c0 | 0.008 | 2.138 | ---NA--- |
| comp108460_c0 | 0.010 | 2.019 | PREDICTED: similar to AGAP001652-PA |
| comp108539_c0 | 0.058 | 2.243 | cathepsin b-like cysteine proteinase-like |
| comp108640_c0 | 0.001 | 2.300 | ---NA--- |
| comp108656_c0 | 0.001 | 3.588 | AGAP002583-PA |
| comp108663_c0 | 0.001 | 2.492 | cg6178 cg6178-pa isoform 1 |
| comp108669_c0 | 0.001 | 2.389 | cuticular protein analogous to peritrophins 3-b precursor |
| comp108734_c0 | 0.010 | 2.105 | PREDICTED: hypothetical protein LOC100575723, partial |
| comp108777_c0 | 0.001 | 2.281 | acid phosphatase-1 |
| comp108825_c1 | 0.037 | 2.565 | ---NA--- |
| comp108826_c0 | 0.032 | 2.142 | peritrophic matrix protein 3 precursor |
| comp108857_c0 | 0.023 | 2.337 | kal-1 protein |
| comp108882_c1 | 0.001 | 2.708 | ---NA--- |
| comp108902_c0 | 0.001 | 2.644 | PREDICTED: similar to AGAP001894-PA |
| comp108962_c1 | 0.001 | 2.615 | ---NA--- |
| comp108997_c1 | 0.001 | 2.385 | atp-binding cassette transporter |
| comp109009_c0 | 0.038 | 2.887 | ---NA--- |
| comp109050_c0 | 0.001 | 3.003 | ---NA--- |
| comp109087_c0 | 0.001 | 2.661 | tan cg12120-pa |
| comp109117_c0 | 0.001 | 2.316 | alcohol dehydrogenase |
| comp109180_c0 | 0.017 | 2.275 | reverse transcriptase, putative |
| comp109194_c0 | 0.008 | 2.061 | isoform a |
| comp109227_c0 | 0.001 | 3.974 | PREDICTED: similar to GA14281-PA |
| comp109230_c0 | 0.001 | 3.124 | AGAP012988-PA |
| comp109270_c0 | 0.001 | 2.305 | prophenoloxidase activating factor |
| comp109291_c0 | 0.001 | 2.413 | PREDICTED: similar to four-jointed protein, putative |
| comp109356_c0 | 0.001 | 6.731 | eukaryotic translation initiation factor 4e binding protein |
| comp109406_c0 | 0.002 | 3.356 | ---NA--- |
| comp109418_c1 | 0.001 | 2.559 | cytochrome p450 monooxygenase |
| comp109449_c0 | 0.001 | 2.015 | ---NA--- |
| comp109498_c0 | 0.001 | 2.713 | ---NA--- |
| comp109522_c0 | 0.001 | 3.706 | PREDICTED: similar to GA14040-PA |
| comp109529_c0 | 0.001 | 2.577 | sodium-dependent phosphate transporter |
| comp109623_c0 | 0.004 | 3.966 | PREDICTED: hypothetical protein |
| comp109630_c0 | 0.004 | 2.109 | PREDICTED: hypothetical protein |
| comp109633_c0 | 0.002 | 2.233 | PREDICTED: similar to putative esterase |
| comp109639_c0 | 0.001 | 2.517 | sodium-dependent phosphate transporter |
| comp109645_c0 | 0.001 | 2.695 | cytochrome p450 |
| comp109709_c1 | 0.001 | 5.467 | cuticular protein |
| comp109774_c0 | 0.001 | 4.200 | PREDICTED: hypothetical protein LOC100574204 |
| comp109791_c1 | 0.001 | 4.851 | uncharacterized protein LOC662961 |
| comp109841_c0 | 0.001 | 2.149 | ---NA--- |
| comp109849_c0 | 0.001 | 2.404 | cg14275 cg14275-pa |
| comp109878_c0 | 0.001 | 2.382 | phosphodiesterase 9 cg32648-pa |
| comp109936_c0 | 0.001 | 8.132 | ---NA--- |
| comp109975_c1 | 0.001 | 2.739 | phosphatidylinositol transfer protein |
| comp109993_c0 | 0.001 | 2.173 | PREDICTED: similar to GA18075-PA |
| comp110056_c0 | 0.001 | 4.191 | tyrosine hydroxylase |
| comp110071_c0 | 0.021 | 2.090 | cg7675 cg7675-pb |
| comp110160_c0 | 0.001 | 2.685 | cg17664 cg17664-pb |
| comp110161_c0 | 0.001 | 3.260 | antennal-enriched xdp-glycosyltransferase |
| comp110229_c0 | 0.009 | 2.575 | ---NA--- |
| comp110251_c0 | 0.001 | 2.544 | cg9119 cg9119-pa |
| comp110258_c0 | 0.001 | 3.682 | cg6084 cg6084-pa |
| comp110286_c1 | 0.001 | 3.559 | equilibrative nucleoside |
| comp110295_c0 | 0.001 | 2.906 | cuticular protein ld-cp3 |
| comp110333_c2 | 0.001 | 2.423 | PREDICTED: similar to AGAP011736-PA |
| comp110336_c0 | 0.001 | 2.171 | PREDICTED: similar to AGAP002198-PA |
| comp110381_c0 | 0.002 | 3.000 | ---NA--- |
| comp110386_c0 | 0.001 | 2.663 | ---NA--- |
| comp110444_c0 | 0.007 | 2.385 | probable c-5 sterol desaturase-like |
| comp110568_c0 | 0.001 | 2.221 | cuticle protein cp5 |
| comp110572_c0 | 0.001 | 5.599 | cuticular protein precursor |
| comp110592_c0 | 0.001 | 4.551 | PREDICTED: similar to copia-type polyprotein, putative |
| comp110599_c0 | 0.001 | 2.241 | propionyl- carboxylase alpha mitochondrial precursor (pccase subunit alpha) (propanoyl- :carbon dioxide ligase subunit alpha) |
| comp110625_c0 | 0.001 | 2.241 | ---NA--- |
| comp110686_c0 | 0.023 | 2.152 | carrier protein |
| comp110718_c0 | 0.001 | 8.646 | alcohol dehydrogenase |
| comp110766_c0 | 0.013 | 2.083 | ---NA--- |
| comp110802_c1 | 0.024 | 2.394 | endonuclease and reverse transcriptase-like protein |
| comp110813_c0 | 0.001 | 2.745 | glucosyl glucuronosyl transferases |
| comp110852_c0 | 0.001 | 2.159 | PREDICTED: similar to AGAP008839-PA |
| comp110885_c0 | 0.001 | 3.478 | amino acid transporter |
| comp110906_c0 | 0.001 | 8.107 | PREDICTED: hypothetical protein |
| comp110912_c0 | 0.004 | 2.212 | cytochrome p450 monooxigenase cyp4q3 |
| comp110933_c0 | 0.001 | 2.889 | AGAP013476-PA |
| comp110935_c0 | 0.001 | 14.210 | pancreatic lipase |
| comp110977_c0 | 0.001 | 2.629 | lysosomal thiol reductase ip30 precursor |
| comp110990_c0 | 0.014 | 2.068 | PREDICTED: similar to AGAP010241-PA |
| comp110991_c0 | 0.001 | 2.680 | AGAP001009-PA |
| comp110997_c1 | 0.001 | 2.715 | cg3106 cg3106-pa |
| comp111132_c0 | 0.001 | 2.320 | PREDICTED: similar to GA12046-PA |
| comp111171_c0 | 0.042 | 2.160 | chitinase 3 |
| comp111182_c1 | 0.001 | 3.673 | copper-zinc superoxide dismutase |
| comp111205_c3 | 0.030 | 2.142 | ---NA--- |
| comp111326_c0 | 0.001 | 3.533 | transposable element p transposase (p-element transposase) |
| comp111372_c0 | 0.001 | 3.052 | PREDICTED: similar to AGAP002559-PA |
| comp111380_c0 | 0.002 | 2.033 | PREDICTED: similar to Hibadhb |
| comp111467_c0 | 0.001 | 2.118 | cuticular protein analogous to peritrophins 3-a1 precursor |
| comp111553_c1 | 0.047 | 2.410 | ---NA--- |
| comp111560_c0 | 0.001 | 2.058 | cuticular precursor |
| comp111573_c0 | 0.001 | 2.403 | ---NA--- |
| comp111590_c0 | 0.001 | 3.088 | cg3625 cg3625-pc |
| comp111616_c0 | 0.001 | 2.952 | PREDICTED: similar to predicted protein |
| comp111617_c0 | 0.001 | 2.825 | ---NA--- |
| comp111631_c0 | 0.001 | 2.772 | ---NA--- |
| comp111641_c0 | 0.041 | 2.760 | AGAP002387-PA |
| comp111653_c0 | 0.001 | 4.176 | PREDICTED: similar to GA13362-PA |
| comp111660_c0 | 0.007 | 2.316 | ---NA--- |
| comp111667_c1 | 0.001 | 2.825 | cg1213 cg1213-pa |
| comp111672_c0 | 0.001 | 2.660 | PREDICTED: similar to AGAP012156-PA |
| comp111691_c1 | 0.001 | 3.253 | cytochrome p450 monooxygenase |
| comp111701_c1 | 0.001 | 2.214 | cg5044 cg5044-pa |
| comp111719_c0 | 0.001 | 3.256 | cationic amino acid transporter |
| comp111753_c0 | 0.001 | 2.522 | PREDICTED: similar to conserved hypothetical protein |
| comp111768_c1 | 0.001 | 3.036 | juvenile hormone-inducible |
| comp111824_c0 | 0.001 | 2.608 | dimethylaniline monooxygenase |
| comp111867_c0 | 0.001 | 4.083 | ---NA--- |
| comp111874_c0 | 0.001 | 2.113 | beta 1-like 2 |
| comp111900_c0 | 0.001 | 2.123 | monoacylglycerol lipase abhd12-like isoform 1 |
| comp111910_c0 | 0.004 | 2.210 | PREDICTED: similar to AGAP006427-PA |
| comp111912_c0 | 0.001 | 3.577 | cytochrome p450 9z4 |
| comp111916_c1 | 0.001 | 2.822 | uncharacterized protein LOC662961 |
| comp111951_c0 | 0.001 | 2.209 | ---NA--- |
| comp111971_c0 | 0.001 | 2.195 | PREDICTED: similar to AGAP001553-PA |
| comp111980_c0 | 0.001 | 3.758 | PREDICTED: hypothetical protein LOC100570299 |
| comp111989_c0 | 0.001 | 2.159 | ribosomal protein l4e |
| comp112010_c0 | 0.001 | 2.021 | cg15786 cg15786-pa |
| comp112148_c0 | 0.001 | 3.299 | PREDICTED: similar to cystathionine-beta-synthase |
| comp112222_c0 | 0.001 | 2.980 | juvenile hormone-inducible |
| comp112295_c0 | 0.001 | 3.378 | tpr repeat-containing protein c9orf52 |
| comp112419_c0 | 0.001 | 5.354 | PREDICTED: hypothetical protein |
| comp112427_c1 | 0.001 | 2.731 | PREDICTED: similar to F28G4.5 |
| comp112459_c0 | 0.001 | 3.604 | ventral nervous system defective |
| comp112465_c0 | 0.001 | 2.687 | phenylalanine hydroxylase |
| comp112482_c0 | 0.001 | 12.77 | ---NA--- |
| comp112509_c0 | 0.003 | 2.158 | ---NA--- |
| comp112538_c1 | 0.049 | 2.586 | thymus-specific serine protease |
| comp112604_c0 | 0.001 | 4.063 | PREDICTED: similar to GA19585-PA |
| comp112622_c0 | 0.001 | 4.079 | PREDICTED: similar to AGAP005515-PA |
| comp112640_c0 | 0.001 | 2.189 | xdp-n-acetylglucosamine pyrophosphorylase 1 |
| comp112652_c0 | 0.001 | 4.071 | agap000696-pa isoform 1 |
| comp112658_c0 | 0.003 | 2.142 | ---NA--- |
| comp112685_c0 | 0.001 | 5.663 | AGAP005332-PC |
| comp112720_c0 | 0.001 | 2.458 | seven in absentia 1b |
| comp112723_c0 | 0.001 | 2.472 | argininosuccinate synthetase |
| comp112725_c0 | 0.001 | 2.063 | cg6847 cg6847-pa |
| comp112737_c0 | 0.001 | 2.067 | PREDICTED: similar to AGAP010734-PA |
| comp112759_c0 | 0.001 | 3.163 | PREDICTED: similar to AGAP000973-PA |
| comp112915_c0 | 0.001 | 2.040 | antennal-enriched xdp-glycosyltransferase |
| comp112968_c0 | 0.001 | 2.540 | glucosyl glucuronosyl transferases |
| comp113028_c1 | 0.001 | 2.497 | aldo-keto reductase |
| comp113083_c0 | 0.001 | 3.186 | ---NA--- |
| comp113105_c0 | 0.001 | 2.939 | ---NA--- |
| comp113157_c0 | 0.013 | 2.331 | ---NA--- |
| comp113238_c1 | 0.001 | 2.452 | cathepsin b |
| comp113270_c0 | 0.001 | 2.065 | AGAP007074-PA |
| comp113327_c2 | 0.001 | 2.672 | tyrosine aminotransferase |
| comp113360_c0 | 0.001 | 2.448 | equilibrative nucleoside transporter 1 cg11907-pa |
| comp113362_c0 | 0.001 | 2.409 | cg3999 cg3999-pa |
| comp113364_c0 | 0.001 | 4.744 | PREDICTED: similar to AGAP003782-PA |
| comp113369_c0 | 0.001 | 2.104 | sugar transporter |
| comp113433_c1 | 0.001 | 2.177 | conserved hypothetical protein |
| comp113458_c0 | 0.001 | 2.889 | PREDICTED: similar to conserved hypothetical protein |
| comp113504_c0 | 0.001 | 2.674 | ---NA--- |
| comp113507_c0 | 0.001 | 2.182 | lipase 3 |
| comp113528_c0 | 0.019 | 2.228 | ---NA--- |
| comp113542_c1 | 0.001 | 2.539 | scavenger receptor acting in neural tissue and majority of rhodopsin is absent cg12789-pb |
| comp113545_c0 | 0.001 | 2.015 | PREDICTED: similar to AGAP004793-PA |
| comp113584_c0 | 0.001 | 2.259 | PREDICTED: similar to GA18316-PA |
| comp113592_c0 | 0.001 | 3.077 | lysosomal acid lipase |
| comp113595_c0 | 0.001 | 2.010 | abc transporter |
| comp113607_c1 | 0.001 | 2.102 | inebriated protein |
| comp113610_c0 | 0.001 | 2.503 | PREDICTED: hypothetical protein LOC100575357 |
| comp113625_c1 | 0.001 | 7.566 | bifunctional protein fold |
| comp113682_c0 | 0.001 | 2.385 | choline ethanolamine kinase |
| comp113703_c0 | 0.001 | 2.318 | ---NA--- |
| comp113705_c0 | 0.001 | 2.319 | ---NA--- |
| comp113764_c0 | 0.001 | 2.443 | sodium-dependent phosphate transporter |
| comp113845_c2 | 0.001 | 2.778 | kruppel-homolog 1 |
| comp113862_c2 | 0.057 | 2.050 | ---NA--- |
| comp113948_c0 | 0.001 | 4.702 | PREDICTED: similar to Luciferase |
| comp113982_c0 | 0.005 | 2.017 | PREDICTED: hypothetical protein LOC100569635 |
| comp114026_c0 | 0.001 | 2.669 | glutathione synthetase |
| comp114040_c0 | 0.007 | 2.345 | ---NA--- |
| comp114049_c0 | 0.010 | 2.477 | PREDICTED: hypothetical protein LOC100570299 |
| comp114062_c0 | 0.036 | 2.000 | timeless isoform b |
| comp114076_c0 | 0.001 | 2.224 | ornithine decarboxylase |
| comp114081_c0 | 0.001 | 2.722 | PREDICTED: similar to alpha-esterase |
| comp114159_c0 | 0.001 | 2.611 | ---NA--- |
| comp114163_c1 | 0.001 | 2.574 | pxphosphoserine phosphatase |
| comp114166_c0 | 0.001 | 2.304 | PREDICTED: similar to AGAP008487-PA |
| comp114174_c0 | 0.001 | 2.251 | PREDICTED: similar to 4-nitrophenylphosphatase |
| comp114247_c0 | 0.001 | 2.029 | PREDICTED: similar to alpha-esterase |
| comp114328_c0 | 0.001 | 3.721 | cg32645 cg32645-pb |
| comp114330_c0 | 0.001 | 2.738 | arylsulfatase b |
| comp114338_c0 | 0.001 | 4.968 | ---NA--- |
| comp114343_c0 | 0.014 | 2.257 | PREDICTED: similar to carboxylesterase |
| comp114346_c0 | 0.001 | 2.445 | cytochrome p450 |
| comp114347_c0 | 0.001 | 2.186 | beta-n-acetylglucosaminidase nag3 precursor |
| comp114363_c0 | 0.001 | 2.951 | ---NA--- |
| comp114384_c0 | 0.001 | 2.014 | PREDICTED: similar to AGAP006427-PA |
| comp114489_c0 | 0.036 | 2.369 | ---NA--- |
| comp114525_c2 | 0.001 | 2.976 | PREDICTED: hypothetical protein LOC100573898 |
| comp114565_c1 | 0.001 | 3.448 | PREDICTED: similar to F28G4.5 |
| comp114570_c0 | 0.001 | 2.084 | cg12340 cg12340-pa |
| comp114639_c0 | 0.001 | 2.287 | serine proteinase |
| comp114643_c0 | 0.001 | 2.888 | PREDICTED: similar to AGAP009114-PA |
| comp114684_c0 | 0.001 | 2.254 | zinc finger protein 77 |
| comp114697_c0 | 0.001 | 2.467 | uncharacterized protein LOC663557 |
| comp114714_c0 | 0.001 | 3.032 | PREDICTED: similar to carboxylesterase |
| comp114774_c3 | 0.001 | 2.568 | PREDICTED: similar to trehalase |
| comp114782_c0 | 0.001 | 2.332 | uncharacterized protein LOC662961 |
| comp114842_c0 | 0.001 | 2.903 | knickkopf cg6217- partial |
| comp114861_c0 | 0.001 | 2.397 | ---NA--- |
| comp114870_c0 | 0.001 | 3.201 | PREDICTED: similar to beta-glucosidase |
| comp114950_c0 | 0.014 | 2.106 | saposin- isoform a |
| comp115026_c0 | 0.032 | 3.014 | ---NA--- |
| comp115030_c0 | 0.001 | 3.958 | ---NA--- |
| comp115076_c0 | 0.001 | 2.135 | PREDICTED: hypothetical protein |
| comp115119_c0 | 0.001 | 2.623 | ---NA--- |
| comp115127_c0 | 0.001 | 2.231 | xanthine dehydrogenase |
| comp115141_c0 | 0.014 | 2.230 | PREDICTED: similar to AGAP012043-PA |
| comp115178_c0 | 0.001 | 3.141 | sodium solute symporter |
| comp115194_c0 | 0.001 | 2.833 | sodium solute symporter |
| comp115223_c0 | 0.001 | 2.967 | ---NA--- |
| comp115233_c0 | 0.001 | 2.019 | PREDICTED: similar to AGAP010534-PA |
| comp115284_c0 | 0.057 | 2.244 | ---NA--- |
| comp115309_c0 | 0.020 | 3.509 | cytochrome p450 9z4 |
| comp115600_c0 | 0.001 | 2.066 | conserved hypothetical protein |
| comp115654_c0 | 0.001 | 5.789 | matrix metalloproteinase |
| comp115775_c0 | 0.001 | 2.210 | sodium nucleoside cotransporter |
| comp115807_c0 | 0.001 | 3.375 | PREDICTED: similar to AGAP004961-PA |
| comp115838_c0 | 0.005 | 2.159 | ---NA--- |
| comp115842_c1 | 0.001 | 2.341 | chitinase 2 precursor |
| comp115889_c0 | 0.001 | 3.223 | rna-directed dna polymerase from mobile element jockey-like |
| comp115924_c0 | 0.001 | 2.429 | chitin synthase 1 |
| comp115994_c0 | 0.001 | 3.688 | PREDICTED: similar to AGAP004918-PA |
| comp116046_c1 | 0.001 | 4.351 | equilibrative nucleoside transporter 1 cg11907-pa |
| comp116104_c1 | 0.001 | 2.417 | acid phosphatase 1 |
| comp116113_c0 | 0.001 | 2.462 | PREDICTED: similar to AGAP003785-PA |
| comp116153_c0 | 0.001 | 2.344 | cuticular protein analogous to peritrophins 1-h precursor |
| comp116178_c0 | 0.001 | 3.317 | hypothetical protein Phum_PHXM433170 |
| comp116218_c1 | 0.001 | 2.576 | cuticular protein analogous to peritrophins 3-c5 isoform 2 precursor |
| comp116262_c0 | 0.005 | 2.111 | cg1213 cg1213-pa |
| comp116267_c0 | 0.011 | 2.867 | PREDICTED: similar to alpha-esterase |
| comp116310_c0 | 0.001 | 3.069 | cuticular protein analogous to peritrophins 3-a2 precursor |
| comp116431_c0 | 0.001 | 2.248 | AGAP009200-PA |
| comp116539_c1 | 0.001 | 2.677 | probable galactose-1-phosphate uridylyltransferase-like |
| comp116551_c0 | 0.001 | 2.516 | ---NA--- |
| comp116560_c0 | 0.001 | 2.450 | cytochrome p450 |
| comp116625_c0 | 0.001 | 2.067 | PREDICTED: similar to conserved hypothetical protein |
| comp116671_c0 | 0.001 | 2.503 | ---NA--- |
| comp116743_c1 | 0.001 | 3.198 | lysosomal alpha-mannosidase (mannosidase alpha class 2b member 1) |
| comp116755_c1 | 0.001 | 2.252 | collagen alpha-2 chain |
| comp116782_c0 | 0.001 | 2.928 | glucose dehydrogenase |
| comp116790_c0 | 0.001 | 2.009 | AGAP005223-PA |
| comp116803_c0 | 0.001 | 4.242 | PREDICTED: similar to AGAP008487-PA |
| comp116810_c0 | 0.002 | 2.118 | PREDICTED: similar to GA20668-PA |
| comp116830_c1 | 0.047 | 2.181 | ---NA--- |
| comp116842_c0 | 0.001 | 2.167 | ---NA--- |
| comp116927_c1 | 0.007 | 3.553 | ---NA--- |
| comp116958_c0 | 0.001 | 3.178 | labial |
| comp116970_c0 | 0.001 | 5.788 | forked cg5424-pb |
| comp117027_c0 | 0.001 | 3.114 | cg8709 cg8709-pb |
| comp117070_c0 | 0.001 | 3.860 | transposable element p transposase (p-element transposase) |
| comp117081_c0 | 0.053 | 6.079 | ---NA--- |
| comp117090_c0 | 0.009 | 2.360 | ---NA--- |
| comp117095_c0 | 0.037 | 2.105 | cg10440 cg10440-pa |
| comp117132_c0 | 0.001 | 2.959 | PREDICTED: similar to phosphoribosylformylglycinamidine synthase, putative |
| comp117214_c0 | 0.002 | 2.044 | reverse transcriptase homolog |
| comp117237_c0 | 0.001 | 3.043 | ---NA--- |
| comp117241_c0 | 0.031 | 2.056 | ras-like gtp-binding protein rho1 |
| comp117260_c0 | 0.001 | 5.618 | AGAP013007-PA |
| comp117266_c0 | 0.001 | 2.512 | cuticular protein analogous to peritrophins 3-d1 precursor |
| comp117328_c0 | 0.001 | 2.946 | ---NA--- |
| comp117364_c0 | 0.001 | 2.297 | PREDICTED: hypothetical protein |
| comp117371_c0 | 0.001 | 2.189 | multi drug resistance 50 cg8523-pa |
| comp117423_c0 | 0.001 | 2.157 | amp dependent ligase |
| comp117463_c1 | 0.001 | 3.954 | ---NA--- |
| comp117469_c0 | 0.001 | 2.836 | sugar transporter |
| comp117538_c0 | 0.001 | 2.211 | atp-binding cassette transporter |
| comp117567_c0 | 0.001 | 3.008 | myc proto-oncogene |
| comp117573_c0 | 0.001 | 4.112 | CG11409 |
| comp117595_c0 | 0.001 | 3.674 | cad88c cg3389-pa |
| comp117597_c0 | 0.001 | 2.129 | elongation of very long chain fatty acids protein aael008004-like |
| comp117605_c0 | 0.001 | 2.028 | sphingomyelin phosphodiesterase |
| comp117622_c0 | 0.001 | 2.547 | cg7044 cg7044-pa |
| comp117632_c0 | 0.001 | 2.306 | fibrillin 2 |
| comp117706_c0 | 0.001 | 2.378 | collagen alpha-1 chain-like |
| comp117719_c1 | 0.001 | 3.693 | cuticular protein 100a cg12045-pa |
| comp117821_c0 | 0.001 | 2.423 | atp-binding sub-family c (cftr mrp) member 4 |
| comp117866_c0 | 0.001 | 2.038 | PREDICTED: similar to AGAP006427-PA |
| comp117934_c0 | 0.001 | 4.857 | xanthine dehydrogenase oxidase |
| comp117944_c0 | 0.041 | 2.321 | 4-hydroxyphenylpyruvate dioxygenase |
| comp118021_c0 | 0.001 | 2.064 | atp-binding cassette transporter |
| comp118050_c0 | 0.001 | 3.257 | ---NA--- |
| comp118072_c0 | 0.001 | 2.725 | xanthine dehydrogenase |
| comp118086_c0 | 0.001 | 2.792 | zinc finger bed domain-containing protein 5-like |
| comp118098_c0 | 0.027 | 2.592 | lysosomal thiol reductase ip30 precursor |
| comp118149_c0 | 0.001 | 2.376 | isoform a |
| comp118190_c0 | 0.001 | 2.628 | ---NA--- |
| comp118327_c0 | 0.001 | 3.293 | agap008849-pa isoform 1 |
| comp118337_c0 | 0.001 | 3.606 | hemoglobin c1 polymer |
| comp118393_c0 | 0.001 | 5.051 | PREDICTED: hypothetical protein LOC100162732 |
| comp118465_c0 | 0.001 | 2.380 | PREDICTED: similar to beta-glucosidase |
| comp118479_c0 | 0.001 | 2.376 | xanthine dehydrogenase |
| comp118489_c0 | 0.001 | 2.086 | cg32645 cg32645-pb |
| comp118505_c0 | 0.001 | 2.449 | PREDICTED: similar to AGAP006427-PA |
| comp118539_c1 | 0.001 | 2.070 | dimmed cg8667-pa |
| comp118576_c0 | 0.016 | 2.528 | glycogen synthase |
| comp118656_c0 | 0.001 | 2.457 | cg31116 cg31116-pe |
| comp118672_c0 | 0.001 | 2.212 | PREDICTED: similar to AGAP000521-PA |
| comp118740_c0 | 0.001 | 2.396 | PREDICTED: similar to conserved hypothetical protein |
| comp118791_c0 | 0.001 | 3.126 | cuticular protein precursor |
| comp118800_c0 | 0.001 | 4.298 | agap002830-pa isoform 5 |
| comp118907_c0 | 0.001 | 2.325 | chitinase 7 precursor |
| comp118911_c0 | 0.001 | 2.897 | reverse transcriptase, putative |
| comp118914_c0 | 0.003 | 2.273 | PREDICTED: similar to polyprotein |
| comp118980_c0 | 0.001 | 2.532 | sp1070 cg9138-pa |
| comp118999_c0 | 0.001 | 3.198 | PREDICTED: similar to AGAP006427-PA |
| comp119029_c0 | 0.001 | 2.096 | PREDICTED: similar to AGAP003205-PA |
| comp119069_c1 | 0.015 | 2.248 | conserved hypothetical protein |
| comp119130_c0 | 0.001 | 2.053 | PREDICTED: similar to conserved hypothetical protein |
| comp119203_c1 | 0.001 | 2.067 | ---NA--- |
| comp119236_c0 | 0.001 | 2.293 | glt8d3 protein |
| comp119311_c1 | 0.001 | 2.145 | PREDICTED: similar to AGAP012156-PA |
| comp119317_c0 | 0.001 | 2.620 | PREDICTED: similar to conserved hypothetical protein |
| comp119437_c0 | 0.001 | 2.308 | AGAP004533-PA |
| comp119437_c2 | 0.028 | 2.326 | cathepsin b-like like proteinase |
| comp119545_c0 | 0.001 | 2.268 | glucosyl glucuronosyl transferases |
| comp119576_c0 | 0.001 | 2.115 | elongation of very long chain fatty acids protein 4 |
| comp119576_c1 | 0.053 | 2.727 | ---NA--- |
| comp119617_c1 | 0.001 | 2.746 | juvenile hormone-inducible |
| comp119631_c0 | 0.002 | 2.559 | PREDICTED: hypothetical protein |
| comp119659_c0 | 0.001 | 2.953 | PREDICTED: similar to AGAP003493-PC |
| comp119777_c0 | 0.001 | 2.513 | cathepsin d isoform 1 |
| comp119952_c0 | 0.001 | 7.370 | ---NA--- |
| comp122972_c0 | 0.001 | 4.152 | ---NA--- |
| comp136756_c0 | 0.001 | 2.746 | AGAP003277-PA |
| comp46898_c0 | 0.001 | 6.162 | ---NA--- |
| comp61647_c0 | 0.045 | 3.645 | ---NA--- |
| comp67468_c0 | 0.001 | 6.552 | ---NA--- |
| comp78210_c0 | 0.014 | 3.300 | ---NA--- |
| comp80499_c0 | 0.001 | 3.074 | ccp84ad cg2341-pa |
| comp80899_c0 | 0.016 | 2.205 | ---NA--- |
| comp81107_c0 | 0.001 | 2.862 | ---NA--- |
| comp82445_c0 | 0.006 | 4.783 | multidrug resistance |
| comp85230_c0 | 0.001 | 118.341 | ---NA--- |
| comp88333_c0 | 0.035 | 2.904 | ---NA--- |
| comp88590_c0 | 0.008 | 2.369 | ---NA--- |
| comp89053_c0 | 0.016 | 2.452 | ---NA--- |
| comp89314_c0 | 0.024 | 2.672 | cg30380 cg30380-pa |
| comp91122_c0 | 0.034 | 2.462 | ---NA--- |
| comp92892_c0 | 0.042 | 2.897 | ---NA--- |
| comp92895_c0 | 0.001 | 2.925 | AGAP006261-PA |
| comp93153_c0 | 0.053 | 3.022 | ---NA--- |
| comp93642_c0 | 0.018 | 4.973 | peritrophic matrix protein 3 precursor |
| comp93996_c0 | 0.001 | 3.465 | ---NA--- |
| comp95355_c0 | 0.059 | 3.013 | PREDICTED: similar to AGAP006427-PB |
| comp95983_c0 | 0.009 | 3.319 | PREDICTED: hypothetical protein |
| comp96052_c0 | 0.001 | 2.788 | cuticular protein ld-cp1v1 |
| comp96355_c0 | 0.003 | 2.073 | PREDICTED: hypothetical protein |
| comp96687_c0 | 0.001 | 2.632 | PREDICTED: ovochymase-1-like |
| comp96761_c0 | 0.044 | 2.125 | ---NA--- |
| comp97937_c0 | 0.008 | 2.498 | AGAP004936-PA |
| comp98478_c0 | 0.028 | 2.573 | ---NA--- |
| comp98501_c0 | 0.003 | 3.981 | ---NA--- |
| comp99067_c0 | 0.001 | 3.831 | ---NA--- |
| comp99107_c0 | 0.004 | 2.822 | cuticular protein 62bc cg1919-pa |
| comp99207_c0 | 0.001 | 2.103 | ---NA--- |
| comp99330_c0 | 0.001 | 3.102 | ---NA--- |
| comp99498_c0 | 0.009 | 2.340 | drosulfakinins precursor, putative |
| comp99743_c0 | 0.049 | 2.336 | ---NA--- |
| comp99795_c0 | 0.001 | 7.050 | pro-phenol oxidase subunit 2 |
| comp99854_c0 | 0.001 | 2.942 | ---NA--- |
| comp99927_c0 | 0.003 | 3.862 | ---NA--- |
| comp99940_c0 | 0.027 | 2.559 | cuticular protein 62bc cg1919-pa |
