# Supplementary material for: Characterizing Molecular Mechanisms of Imidacloprid Resistance in Select Populations of Leptinotarsa decemlineata in the Central Sands Region of Wisconsin
Source: PLoS One. 2016 Jan 28;11(1):e0147844. doi: 10.1371/journal.pone.0147844 (PMC4731083; doi:10.1371/journal.pone.0147844)
Supplement: S3 Table — (DOCX) [file pone.0147844.s003.docx]

**Supplementary Table S3**: Enrichment analysis between GO terms from the up-regulated transcripts of the systemic-1 population compared to the whole transcriptome.

| GO Term | Name | FDR | Over/Under  expressed GO term in systemic-1 population |
| --- | --- | --- | --- |
| [GO:0044464](fisherinfo:GO:0044464) | cell part | 0.001 | under |
| [GO:0005623](fisherinfo:GO:0005623) | cell | 0.001 | under |
| [GO:0044260](fisherinfo:GO:0044260) | cellular macromolecule metabolic process | 0.001 | under |
| [GO:0005622](fisherinfo:GO:0005622) | intracellular | 0.001 | under |
| [GO:0044424](fisherinfo:GO:0044424) | intracellular part | 0.002 | under |
| [GO:0044710](fisherinfo:GO:0044710) | single-organism metabolic process | 0.023 | over |
| [GO:0003824](fisherinfo:GO:0003824) | catalytic activity | 0.023 | over |
| [GO:0043170](fisherinfo:GO:0043170) | macromolecule metabolic process | 0.023 | under |
| [GO:0016491](fisherinfo:GO:0016491) | oxidoreductase activity | 0.023 | over |
| [GO:0043226](fisherinfo:GO:0043226) | organelle | 0.023 | under |
| [GO:0043229](fisherinfo:GO:0043229) | intracellular organelle | 0.023 | under |
