# Supplementary material for: Characterizing Molecular Mechanisms of Imidacloprid Resistance in Select Populations of Leptinotarsa decemlineata in the Central Sands Region of Wisconsin
Source: PLoS One. 2016 Jan 28;11(1):e0147844. doi: 10.1371/journal.pone.0147844 (PMC4731083; doi:10.1371/journal.pone.0147844)
Supplement: S4 Table — (DOCX) [file pone.0147844.s004.docx]

**Supplementary Table S4**: Enrichment analysis between GO terms from the up-regulated transcripts of the systemic-3 population compared to the whole transcriptome.

| GO Term | Name | FDR | Over/Under  expressed GO term in systemic-3 population |
| --- | --- | --- | --- |
| [GO:0044464](FisherInfo:GO:0044464) | cell part | 0.001 | under |
| [GO:0005623](FisherInfo:GO:0005623) | cell | 0.001 | under |
| [GO:0005622](FisherInfo:GO:0005622) | intracellular | 0.001 | under |
| [GO:0044424](FisherInfo:GO:0044424) | intracellular part | 0.001 | under |
| [GO:0044260](FisherInfo:GO:0044260) | cellular macromolecule metabolic process | 0.001 | under |
| [GO:0043229](FisherInfo:GO:0043229) | intracellular organelle | 0.001 | under |
| [GO:0043226](FisherInfo:GO:0043226) | organelle | 0.001 | under |
| [GO:0016491](FisherInfo:GO:0016491) | oxidoreductase activity | 0.001 | over |
| [GO:1901564](FisherInfo:GO:1901564) | organonitrogen compound metabolic process | 0.001 | over |
| [GO:0044710](FisherInfo:GO:0044710) | single-organism metabolic process | 0.001 | over |
| [GO:0006030](FisherInfo:GO:0006030) | chitin metabolic process | 0.001 | over |
| [GO:0055114](FisherInfo:GO:0055114) | oxidation-reduction process | 0.001 | over |
| [GO:0004497](FisherInfo:GO:0004497) | monooxygenase activity | 0.001 | over |
| [GO:0043231](FisherInfo:GO:0043231) | intracellular membrane-bounded organelle | 0.001 | under |
| [GO:0043227](FisherInfo:GO:0043227) | membrane-bounded organelle | 0.001 | under |
| [GO:1901071](FisherInfo:GO:1901071) | glucosamine-containing compound metabolic process | 0.001 | over |
| [GO:0006040](FisherInfo:GO:0006040) | amino sugar metabolic process | 0.001 | over |
| [GO:0008061](FisherInfo:GO:0008061) | chitin binding | 0.001 | over |
| [GO:0006022](FisherInfo:GO:0006022) | aminoglycan metabolic process | 0.001 | over |
| [GO:0032991](FisherInfo:GO:0032991) | macromolecular complex | 0.001 | under |
| [GO:0005737](FisherInfo:GO:0005737) | cytoplasm | 0.001 | under |
| [GO:0003824](FisherInfo:GO:0003824) | catalytic activity | 0.001 | over |
| [GO:0005506](FisherInfo:GO:0005506) | iron ion binding | 0.001 | over |
| [GO:0003676](FisherInfo:GO:0003676) | nucleic acid binding | 0.001 | under |
| [GO:0044267](FisherInfo:GO:0044267) | cellular protein metabolic process | 0.001 | under |
| [GO:0043234](FisherInfo:GO:0043234) | protein complex | 0.001 | under |
| [GO:0020037](FisherInfo:GO:0020037) | heme binding | 0.001 | over |
| [GO:0046906](FisherInfo:GO:0046906) | tetrapyrrole binding | 0.001 | over |
| [GO:0006685](FisherInfo:GO:0006685) | sphingomyelin catabolic process | 0.001 | over |
| [GO:0006684](FisherInfo:GO:0006684) | sphingomyelin metabolic process | 0.001 | over |
| [GO:0009308](FisherInfo:GO:0009308) | amine metabolic process | 0.001 | over |
| [GO:0044106](FisherInfo:GO:0044106) | cellular amine metabolic process | 0.001 | over |
| [GO:0044422](FisherInfo:GO:0044422) | organelle part | 0.001 | under |
| [GO:1901565](FisherInfo:GO:1901565) | organonitrogen compound catabolic process | 0.002 | over |
| [GO:0016705](FisherInfo:GO:0016705) | oxidoreductase activity, acting on paired donors, with incorporation or reduction of molecular oxygen | 0.002 | over |
| [GO:0071840](FisherInfo:GO:0071840) | cellular component organization or biogenesis | 0.002 | under |
| [GO:0044446](FisherInfo:GO:0044446) | intracellular organelle part | 0.002 | under |
| [GO:0050896](FisherInfo:GO:0050896) | response to stimulus | 0.003 | under |
| [GO:0009987](FisherInfo:GO:0009987) | cellular process | 0.003 | under |
| [GO:0009395](FisherInfo:GO:0009395) | phospholipid catabolic process | 0.003 | over |
| [GO:0090304](FisherInfo:GO:0090304) | nucleic acid metabolic process | 0.003 | under |
| [GO:0016043](FisherInfo:GO:0016043) | cellular component organization | 0.005 | under |
| [GO:0044444](FisherInfo:GO:0044444) | cytoplasmic part | 0.008 | under |
| [GO:0044281](FisherInfo:GO:0044281) | small molecule metabolic process | 0.008 | over |
| [GO:0005634](FisherInfo:GO:0005634) | nucleus | 0.014 | under |
| [GO:0006520](FisherInfo:GO:0006520) | cellular amino acid metabolic process | 0.014 | over |
| [GO:0042439](FisherInfo:GO:0042439) | ethanolamine-containing compound metabolic process | 0.014 | over |
| [GO:1901616](FisherInfo:GO:1901616) | organic hydroxy compound catabolic process | 0.014 | over |
| [GO:0046164](FisherInfo:GO:0046164) | alcohol catabolic process | 0.014 | over |
| [GO:0009072](FisherInfo:GO:0009072) | aromatic amino acid family metabolic process | 0.014 | over |
| [GO:0050794](FisherInfo:GO:0050794) | regulation of cellular process | 0.014 | under |
| [GO:0042302](FisherInfo:GO:0042302) | structural constituent of cuticle | 0.014 | over |
| [GO:0044707](FisherInfo:GO:0044707) | single-multicellular organism process | 0.014 | under |
| [GO:0009169](FisherInfo:GO:0009169) | purine ribonucleoside monophosphate catabolic process | 0.014 | over |
| [GO:0009158](FisherInfo:GO:0009158) | ribonucleoside monophosphate catabolic process | 0.014 | over |
| [GO:0009128](FisherInfo:GO:0009128) | purine nucleoside monophosphate catabolic process | 0.014 | over |
| [GO:0009125](FisherInfo:GO:0009125) | nucleoside monophosphate catabolic process | 0.014 | over |
| [GO:0006200](FisherInfo:GO:0006200) | ATP catabolic process | 0.014 | over |
| [GO:0006576](FisherInfo:GO:0006576) | cellular biogenic amine metabolic process | 0.017 | over |
| [GO:0030149](FisherInfo:GO:0030149) | sphingolipid catabolic process | 0.020 | over |
| [GO:0046466](FisherInfo:GO:0046466) | membrane lipid catabolic process | 0.020 | over |
| [GO:0044712](FisherInfo:GO:0044712) | single-organism catabolic process | 0.020 | over |
| [GO:0044282](FisherInfo:GO:0044282) | small molecule catabolic process | 0.020 | over |
| [GO:0043170](FisherInfo:GO:0043170) | macromolecule metabolic process | 0.024 | under |
| [GO:0032502](FisherInfo:GO:0032502) | developmental process | 0.027 | under |
| [GO:0019538](FisherInfo:GO:0019538) | protein metabolic process | 0.028 | under |
| [GO:1901615](FisherInfo:GO:1901615) | organic hydroxy compound metabolic process | 0.033 | over |
| [GO:0019752](FisherInfo:GO:0019752) | carboxylic acid metabolic process | 0.033 | over |
| [GO:0005576](FisherInfo:GO:0005576) | extracellular region | 0.035 | over |
| [GO:0051716](FisherInfo:GO:0051716) | cellular response to stimulus | 0.035 | under |
| [GO:0032501](FisherInfo:GO:0032501) | multicellular organismal process | 0.035 | under |
| [GO:0006066](FisherInfo:GO:0006066) | alcohol metabolic process | 0.035 | over |
| [GO:0065007](FisherInfo:GO:0065007) | biological regulation | 0.035 | under |
| [GO:0043436](FisherInfo:GO:0043436) | oxoacid metabolic process | 0.035 | over |
| [GO:0044699](FisherInfo:GO:0044699) | single-organism process | 0.035 | under |
| [GO:0006082](FisherInfo:GO:0006082) | organic acid metabolic process | 0.036 | over |
| [GO:0006665](FisherInfo:GO:0006665) | sphingolipid metabolic process | 0.037 | over |
| [GO:0004767](FisherInfo:GO:0004767) | sphingomyelin phosphodiesterase activity | 0.037 | over |
| [GO:0055085](FisherInfo:GO:0055085) | transmembrane transport | 0.038 | over |
| [GO:1901605](FisherInfo:GO:1901605) | alpha-amino acid metabolic process | 0.042 | over |
| [GO:0007154](FisherInfo:GO:0007154) | cell communication | 0.044 | under |
| [GO:0009055](FisherInfo:GO:0009055) | electron carrier activity | 0.044 | over |
| [GO:0007275](FisherInfo:GO:0007275) | multicellular organismal development | 0.047 | under |
| [GO:0043603](FisherInfo:GO:0043603) | cellular amide metabolic process | 0.050 | over |
